# Co‐Expression of MHC‐II and ANXA1: Mediators of PD‐1/PD‐L1 Therapy Resistance in Breast Cancer

**DOI:** 10.1002/cnr2.70291

**Published:** 2025-07-31

**Authors:** Hao Wang, Ji‐Feng Sun, Chen Wang, Zhan‐Sheng Jiang, Zhong‐Sheng Tong

**Affiliations:** ^1^ Tianjin Medical University Cancer Institute & Hospital, National Clinical Research Center for Cancer Tianjin China; ^2^ Tianjin's Clinical Research Center for Cancer Tianjin China; ^3^ Key Laboratory of Breast Cancer Prevention and Therapy, Tianjin Medical University, Ministry of Education Tianjin China; ^4^ Key Laboratory of Cancer Prevention and Therapy Tianjin China; ^5^ Tianjin Cancer Hospital Airport Hospital Tianjin China

**Keywords:** ANXA1, differential gene expression, immunotherapy, Mendelian randomization, MHC‐II, PD‐1/PD‐L1 resistance, single‐cell sequencing, triple‐negative breast cancer

## Abstract

**Background:**

Triple‐negative breast cancer (TNBC) presents significant treatment challenges and poor prognosis. While immune checkpoint blockade (ICB) therapy shows promise, patient responses vary widely, highlighting the urgent need for reliable biomarkers to predict efficacy and guide treatment decisions.

**Aims:**

This study aims to investigate the role of Major Histocompatibility Complex class II (MHC‐II) expression in breast cancer, specifically focusing on its impact on immune evasion, tumor metastasis, and immunotherapy efficacy. The objective is to emphasize the necessity of targeted research in order to enhance therapeutic strategies for TNBC.

**Methods:**

We employed Limma for conducting differential expression analysis, clusterProfiler for performing GO and KEGG pathway enrichment analyses, and Mendelian randomization analyses utilizing data from the UK Biobank and GEO data sets. Single‐cell sequencing data were analyzed using Scanpy and CellTypist, where UMAP, PCA, and the Leiden algorithm were applied to explore cellular heterogeneity as well as gene expression profiles.

**Results:**

We observed significant differential gene expression between MHC‐II‐high and MHC‐II‐low hematopoietic stem cells, which has an impact on immune responses and cancer pathways, particularly in TNBC. Mendelian randomization analysis identified key genes associated with breast cancer risk and PD‐L1 status. Additionally, ANXA1 was significantly decreased in expression in breast cancer tissues compared to normal tissues and demonstrated increased expression in nonresponders to PD‐1/PD‐L1 therapies in TNBC patients, suggesting its potential involvement in immunotherapy resistance despite lacking a direct correlation with overall survival rates.

**Conclusion:**

The findings of this study highlight the potential role of ANXA1 in mediating resistance to PD‐1/PD‐L1 therapy in breast cancer, which is associated with MHC‐II expression. ANXA1 could serve as both a predictive marker for treatment resistance and a therapeutic target to enhance the efficacy of immunotherapy.

## 1 Introduction

Breast cancer is a highly prevalent and severe disease affecting women worldwide, with triple‐negative breast cancer (TNBC) being an especially challenging subtype due to its resistance to treatment [1]. Despite advances in targeted therapies, TNBC patients still have poor prognoses, necessitating alternative therapeutic strategies. Recent evidence highlights the crucial role of the immune system in preventing and eradicating cancer, with immune checkpoint blockade (ICB) therapies showing promise for various cancers, including TNBC. However, patient response variability to ICB therapy underscores the need for reliable biomarkers that can predict treatment outcomes and guide therapeutic decisions.

In this context, MHC‐II has emerged as a pivotal component in the realm of anticancer immunity. Traditionally acknowledged for their involvement in antigen presentation and activation of CD4^+^ T cells, MHC‐II molecules play an indispensable role in initiating and sustaining tumor immunity. A recent study revealed a significant correlation between the presence of MHC‐II+ cancer cells and the effectiveness of ICB therapy among TNBC patients, thereby suggesting that the expression of MHC‐II in tumor cells can serve as a marker for therapeutic response [2]. Furthermore, a study has demonstrated that MHC‐II expression in cancer cells plays a crucial role in promoting immune tolerance within the tumor microenvironment. This is achieved through the expansion of regulatory T cells (Tregs) and inhibition of effector T cell function, ultimately facilitating tumor metastasis to lymph nodes [3]. This dual nature of MHC‐II in both antitumor immunity and immune evasion suggests that targeting altered MHC‐II expression could potentially serve as a novel therapeutic strategy for breast cancer. Given the importance of MHC‐II in modulating the immune response within the tumor microenvironment, recent studies have enhanced our comprehension of its involvement in breast cancer pathology. Moreover, it has been found that MHC‐II/CD4‐dependent metastasis further exacerbates this phenomenon [4].

This finding emphasizes the important role of MHC‐II in tumor immunity and metastasis, suggesting that altered expression of C/EBPβ may influence levels of MHC‐II, thereby potentially affecting subsequent proliferation of immune responses by tumors. The study revealed that the expression of tumor‐specific MHC‐II could serve as a predictive factor for the efficacy of anti‐PD‐1/L1 therapy in patients with HER2‐negative primary breast cancer [5]. Furthermore, the study identified MHC‐II inducers that significantly increase MHC‐II expression in breast cancer cells, thus inducing immune responses. Detection and anticancer effects between cancer physiology and the immune system are enhanced by highlighting the links. This study offers a novel approach to cancer therapy through synthetic approaches targeting mutants, which increase MHC‐II expression and regulate antitumor immunity [6].

Moreover, the studies [7, 8] have further enhanced our comprehension of the role of MHC‐II in the tumor microenvironment. These studies have demonstrated that the expression of MHC‐II on nontumor cells, such as neutrophils and fibroblasts, can impact tumor progression, metastasis size, and immune response. These findings highlight the intricate nature of tumor–immune interactions and emphasize the significance of a comprehensive comprehension of MHC‐II's multifaceted role in cancer biology. In light of these discoveries, further investigation is warranted regarding the correlation between MHC‐II and breast cancer, particularly within the realm of immunotherapy. Gaining an understanding of the mechanisms by which MHC‐II expression influences both the tumor microenvironment and the immune response to cancer is pivotal for developing more efficacious and targeted therapies for TNBC and other subtypes of breast cancer.

The objective of this investigation was to further explore the correlation between MHC‐II expression and breast cancer, with a specific focus on its potential as both a therapeutic target and biomarker for immune response.

## 2 Methods

### 2.1 Data Acquisition and Processing

In our study, we obtained count data from the GEO data set GSE211206 (https://www.ncbi.nlm.nih.gov/geo/) and employed the Limma package [9] in R to identify differentially expressed genes (DEGs) between MHC‐II‐high and MHC‐II‐low hematopoietic stem cells (HSCs) isolated from middle‐aged mice. The information, publicly released on August 8, 2023, aims to analyze transcriptome data of this specific cell population primarily derived from 12‐month‐old mice, with a focus on understanding the role of MHC‐II in HSCs. We selected 12‐month‐old mice as a model because they better reflect the state of HSCs in adult breast cancer patients, providing a more physiologically relevant model for studying the immunomodulatory functions of MHC‐II in the tumor microenvironment. We identified DEGs based on an adjusted *p* value (adj.*p*.Val) < 0.05 and a log fold change (logFC) > 1 for upregulated genes or < −1 for downregulated genes.

The data set ukb‐b‐16 890 was utilized for conducting Mendelian randomization (MR) analyses related to breast cancer. It originates from the UK Biobank [10], a large‐scale biomedical database and research resource containing genetic, lifestyle, and health information from half a million UK participants. For the year 2018, the data set focuses on a binary classification representing the presence or absence of breast cancer, covering both male and female participants of European ancestry. It includes data from 10 303 cases (individuals diagnosed with breast cancer) and 452 630 controls (individuals without breast cancer), making up a total sample size of 462 933 individuals. The genetic data encompasses 9 851 867 single‐nucleotide polymorphisms (SNPs), analyzed under the build HG19/GRCh37 of the human genome.

The second data set utilized for the MR analysis focuses on PD‐L1 levels and is referenced as ebi‐a‐GCST90000492. This data set was compiled with the aim of investigating the genetic determinants of PD‐L1 expression levels, which play a crucial role in immune response and have significant implications for cancer therapies, particularly in immunotherapy. This research was led by author Hillary RF. Although no specific consortium or ontology is associated with this data set, it plays a pivotal role in comprehending the genetic foundations of PD‐L1 expression [11]. The study encompasses a European population, without specifying participant genders. The sample size of this particular data set is relatively small, including 982 individuals. Despite this limitation, an extensive number of SNPs, totaling 7 883 829 were analyzed. This comprehensive genetic data enables a thorough investigation into the genetic factors influencing PD‐L1 levels.

This data set, which is a part of BioProject PRJNA1032700 and falls under GSE246613, investigates the response of TNBC to pembrolizumab and radiation therapy [12]. It comprises single‐cell RNA‐sequencing and spatial proteomics data obtained from pre‐ and post‐treatment biopsies. The study classifies tumors into responders and nonresponders based on immune infiltration and therapy‐induced changes, with the aim of identifying patients who benefit from immunotherapy alone or require additional treatments. The data set facilitates an in‐depth examination of cellular behavior in nonresponsive samples to pembrolizumab.

### 2.2 GO and KEGG Pathway Enrichment Analysis

After conducting the differential expression analysis, we proceeded with comprehensive signal pathway enrichment analyses using the R package clusterProfiler. This encompassed gene ontology (GO) enrichment in three primary categories: biological processes (BP), cellular components (CC), and molecular functions (MF), as well as Kyoto Encyclopedia of Genes and Genomes (KEGG) pathway analysis. We established a significance threshold at an adjusted *p* value below 0.05 to identify meaningful biological insights. To visually represent the findings, we utilized the dotplot visualization tool to present the top 10 enriched pathways across GO‐BP, GO‐CC, and GO‐MF categories, along with the top 10 KEGG pathways. This visualization facilitates comprehension of the most significant altered biological functions and components between two comparative groups, which can be considered as MHC‐II‐high vs. MHC‐II‐low HSCs for illustrative purposes; however, specific groups would depend on the context of the study.

### 2.3 Visualization of DEGs Within Key KEGG Pathways

In this research, the R packages “pathway” and “ggKEGG” were specifically employed to construct comprehensive signal pathway diagrams for the top five KEGG pathways identified. These diagrams offer a detailed depiction of the significantly affected biological processes, utilizing log fold change (logFC) data to indicate both the magnitude and direction of gene expression alterations. The “pathway” package facilitates visual representation of these changes, thereby enhancing our comprehension of their biological implications. Simultaneously, the “ggKEGG” package incorporates adjusted *p* value data, emphasizing the statistical significance of gene expression modifications within each identified pathway. Through this analytical and visual approach, we obtain a comprehensive overview of the cellular and molecular foundations that distinguish between the comparative groups under investigation.

### 2.4 Analyzing Pathway Variations: GSEA in MHC‐II Divergent HSCs

In this study, we conducted a gene set enrichment analysis (GSEA) to investigate the differential biological pathways between MHC‐II‐high and MHC‐II‐low HSCs. The GSEA was performed using the ReactomePA package, specifically designed for Reactome pathway analysis [13]. The following commands were executed: The “gsePathway” function was applied to our gene list with the parameters set as follows: “pvalueCutoff = 1” and “pAdjustMethod = BH.” The “pvalueCutoff = 1” setting allowed us to consider all pathways regardless of their initial unadjusted *p* values, providing a comprehensive view of potential biological differences. To control the false discovery rate and ensure robustness of pathway enrichment results, we used the Benjamini–Hochberg (BH) method for adjusting *p* values. Subsequently, in order to facilitate the interpretation of results, we used the “setReadable” function to convert the gene identifiers in the GSEA results to human‐readable gene names. This conversion was achieved by specifying the “OrgDb = org.Hs.eg.db” parameter, which corresponds to the human gene database, and setting “keyType = ENTREZID” to indicate the type of gene identifiers used in our list. The initial results were then extracted and converted into a dataframe for further analysis and visualization by using “head(as.data.frame(GSEA)),” which provided an overview of the top enriched pathways in our data set.

### 2.5 Exploring Genetic Associations With MR Analysis

In this investigation, to maximize the enhancement of potential DEGs for exploring the associations with breast cancer and PD‐L1 levels, we augmented our analysis by utilizing the edgeR package to extract additional DEGs [14]. These newly identified DEGs, combined with those previously identified through the Limma package, constituted an enhanced data set for our subsequent MR analysis. The R package gwasrapidd [15] was employed to extract SNPs associated with these comprehensive sets of DEGs, differentiated between MHC‐II‐high and MHC‐II‐low categories. These SNPs were utilized as instrumental variables in our Summary‐data‐based MR analysis, with the aim of uncovering potential causal relationships. For the MR analyses, we utilized the TwoSampleMR package [16], employing exposure data derived from the UK Biobank project's breast cancer patient data set. Our MR analysis was comprehensive, incorporating several MR methods, including MR Egger, weighted median, inverse variance weighted (IVW), simple mode, and weighted mode. These diverse analytical approaches enabled us to robustly assess the potential causal relationships between genetic variations (reflected by SNPs) associated with MHC‐II‐related DEGs and the outcome variables of breast cancer and PD‐L1 levels. Moreover, this study did not merely investigate the collective influence of all MHC‐II‐related DEGs on breast cancer and PD‐L1 levels but also examined the individual contributions of different DEGs.

Several key parameters are used with the TwoSampleMR package throughout the different stages of data preprocessing, analysis, and visualization:

– Sample size, SNP count: These are basic parameters indicating the scale of our study, affecting the power and resolution of the MR analysis.
– pval.exposure and pval.outcome: In data filtering stages, these thresholds (such as “< 5e−08” for significant SNPs) are crucial for defining what is considered a significant association in both exposure and outcome data sets.
– clump_kb, clump_r2: In the “ld_clump” function, these parameters define the window size (in kilobases) and the R‐squared threshold for LD clumping, crucial for identifying independent genetic variants.
– plink_bin: This specifies the path to the PLINK binary, essential for executing LD clumping [17].
– Harmonization: The “harmonise_data” function adjusts exposure and outcome data for compatibility, ensuring that allele definitions match between data sets.
– MR methods: In the script, different MR methods such as MR Egger, weighted median, and IVW are mentioned. These methods are crucial for the MR analysis, each offering different assumptions and sensitivity to pleiotropy and biases.
– Visualization tools: Functions like “mr_scatter_plot,” “mr_forest_plot,” and “mr_funnel_plot” are used for visual analysis, helping to interpret the results and assess the validity and homogeneity of the instrumental variables.

### 2.6 Methodology for Single‐Cell Sequencing Data Analysis

In this study, we extracted PD1 inhibitor‐treated samples from the GSE246613 PembroRT immune data set in h5ad format to: (1) identify the DEGs between complete response (pCR = R) and no response (pCR = NR) samples; (2) focus on the non‐responding (pCR = NR) samples treated with PD1 inhibitors for subsequent single‐cell sequencing analysis. We specifically targeted DEGs previously validated by MR as being associated with breast cancer risk and PD‐L1 status.

Python packages such as Scanpy [18] were utilized in this study for cell filtering, gene filtering, normalization, log transformation, and identification of highly variable genes. Important parameters considered include minimum gene counts, mitochondrial DNA content, and quality control metrics. Visualization tools like UMAP, PCA, and violin plots were employed to analyze cellular heterogeneity and marker expression. The study specifically analyzed PD1‐treated samples from the GSE246613 data set while focusing on DEGs related to breast cancer risk and PD‐L1 status. Key parameters considered in this single‐cell sequencing analysis included:

– Minimum genes per cell: 200
– Minimum cells per gene: 3
– Mitochondrial gene content filtering: cells with < 5% mitochondrial genes
– Gene expression normalization: total counts per cell scaled to 10,000
– Dimensionality reduction: PCA, using the top 40 principal components
– Neighbors: calculating using 10 neighbors
– Clustering: Leiden algorithm
– Differential expression: methods including *t*‐test, Wilcoxon rank‐sum, and logistic regression

Visualization tools used were UMAP for data embedding, violin plots for gene expression, and scatter plots for mitochondrial content vs. total counts. Additionally, cell type annotations were enhanced using the CellTypist tool [19].

### 2.7 Statistical Methods

To ensure robust and reliable data interpretation, we employed a series of statistical methods tailored to the specific needs of each data set. The DEGs between MHC‐II‐high and MHC‐II‐low HSCs were identified using the Limma package in R, with a significance threshold of an adjusted *p* value (adj.*p*.Val) < 0.05 and a log fold change (logFC) > 1 or < −1. MR analysis was conducted using the TwoSampleMR R package, incorporating methods such as MR Egger, weighted median, IVW, simple mode, and weighted mode, with a significance threshold of *p* value < 5e−08 for SNPs. For single‐cell RNA‐sequencing data, differential expression was assessed using *t*‐tests, Wilcoxon rank‐sum tests, and logistic regression.

## 3 Results

### 3.1 DEGs in MHC‐II Subgroups: Insights From Limma Analysis

**FIGURE 1:**
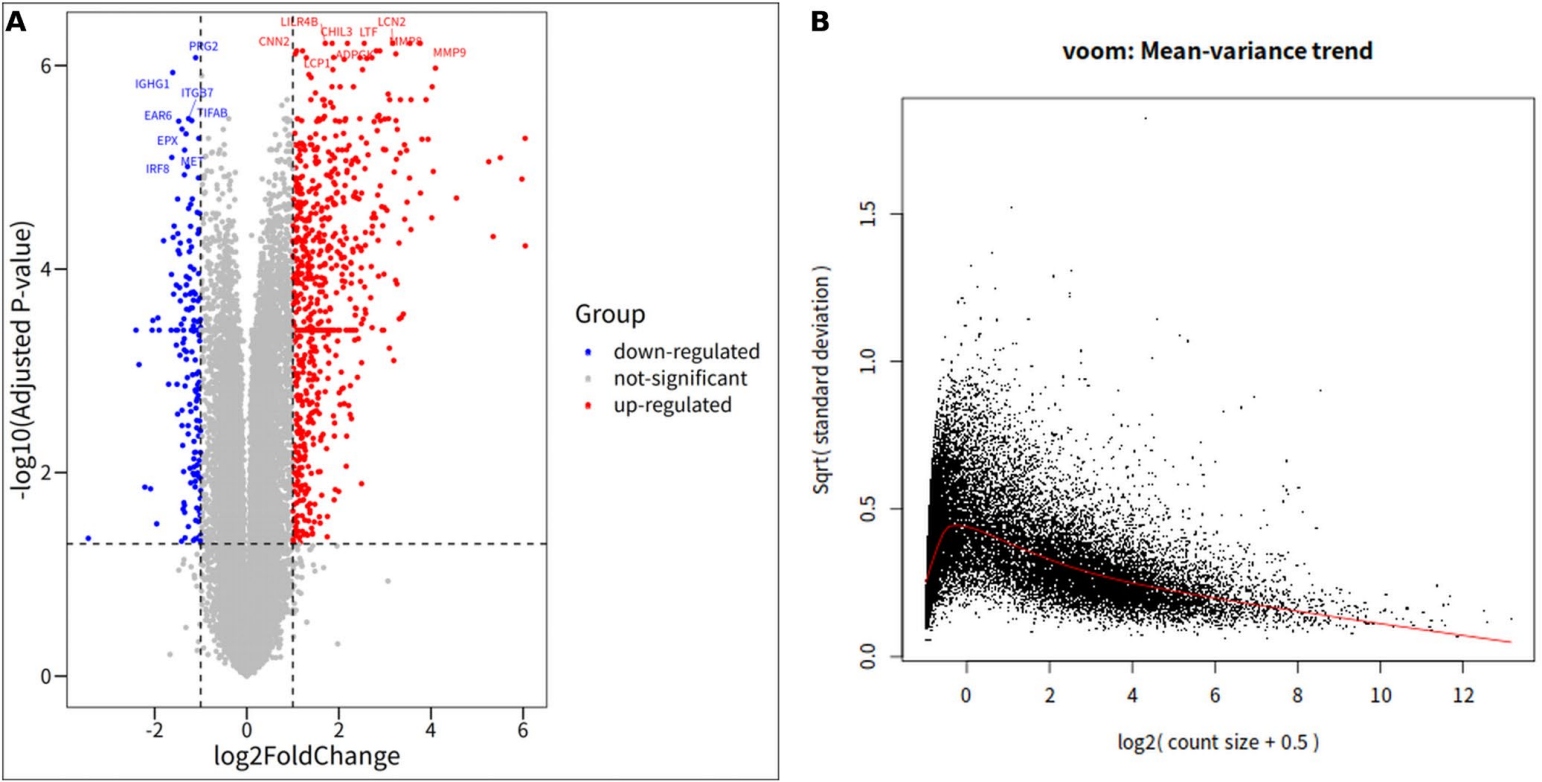
Volcano plot showing differentially expressed genes (DEGs) between MHC‐II‐high and MHC‐II‐low HSCs. DEGs were identified using Limma with an adjusted *p* value < 0.05 and a logFC > 1 or < −1. (A) Volcano plot of DEGs between MHC‐II subgroups. (B) Trend of average gene expression across DEGs.

This analysis, conducted using the Limma package, as shown in Figure 1A, resulted in the identification of 718 upregulated and 174 downregulated DEGs. Additionally, it was observed that the variance decreases with an increase in average gene expression levels, as depicted by the downward trend of the red line amidst the black data points (Figure 1B). Among the top 20 DEGs listed in Table 1, Lactotransferrin (LTF) exhibited the highest upregulation with a logFC value of 2.5505. This significant upregulation indicates its crucial role in regulating iron levels and possessing antimicrobial and anti‐inflammatory properties. Another noteworthy gene, matrix metallopeptidase 8 (MMP8), exhibited a logFC of 3.5416, indicating its critical role in the degradation of proteins within the extracellular matrix. Similarly, Lipocalin 2 (LCN2) and matrix metallopeptidase 9 (MMP9) were significantly upregulated with logFC values of 3.172 and 3.7653, respectively, suggesting their involvement in the transportation and breakdown of extracellular matrix proteins. Notably, genes such as CHIL3, LILR4B, and MMP9 associated with inflammation, immune cell activity, and tissue remodeling were also markedly upregulated, emphasizing the potential immunological functions of these genes in HSCs. Additionally, Adenosine diphosphate‐dependent glucokinase (ADPGK) and Lymphocyte cytosolic protein 1 (LCP1) were identified as key players in metabolic processes and T cell activation, exhibiting significant upregulation in MHC‐II‐high cells. In contrast, Proteoglycan 2 (PRG2), with a logFC of −1.1105, was the only gene among the top 20 DEGs that showed downregulation. This suggests that its involvement in cell–cell adhesion and extracellular matrix organization is less pronounced in MHC‐II‐high compared to MHC‐II‐low HSCs. This differential expression profile, particularly the significant upregulation of genes involved in immune response, inflammation, and cellular metabolism, offers valuable insights into the functional disparities between MHC‐II‐high and MHC‐II‐low HSCs and emphasizes the potential regulatory mechanisms of MHC‐II in the aging hematopoietic system.

**TABLE 1:** Top 20 DEGs in MHC‐II high vs. low hematopoietic stem cells.

Symbol	Annotation	LogFC	Average expression	*t*	*p*	Adjusted *p*	*B*
LTF	Lactotransferrin, a protein that helps regulate iron levels and has antimicrobial and anti‐inflammatory properties.	2.550508063	10.34685245	205.5490645	5.14E−11	6.06E−07	16.62772305
CHIL3	Chitinase‐like protein 3, a protein that plays a role in inflammation and tissue remodeling.	1.853699407	11.87806224	189.1772899	7.79E−11	6.06E−07	16.12485594
LILR4B	Leukocyte immunoglobulin‐like receptor 4B, a protein that regulates immune cell activity.	1.704720337	9.326012353	184.4250112	8.85E−11	6.06E−07	16.02388173
MMP8	Matrix metallopeptidase 8, an enzyme that breaks down proteins in the extracellular matrix.	3.541623464	9.503434341	176.6637622	1.10E−10	6.06E−07	15.78796815
LCN2	Lipocalin 2, a protein that binds and transports small hydrophobic molecules.	3.171562634	9.844366299	173.7696508	1.19E−10	6.06E−07	15.78133789
MMP9	Matrix metallopeptidase 9, an enzyme that breaks down proteins in the extracellular matrix.	3.765306931	8.402672935	168.7319476	1.38E−10	6.06E−07	15.30724099
ADPGK	Adenosine diphosphate‐dependent glucokinase, an enzyme that catalyzes the phosphorylation of glucose.	2.186191032	6.703465878	169.4566547	1.35E−10	6.06E−07	14.78165059
LCP1	Lymphocyte cytosolic protein 1, a protein that plays a role in T cell activation.	1.211480021	8.826837919	149.0169664	2.57E−10	7.18E−07	15.01195413
CNN2	Calponin 2, a protein that regulates smooth muscle contraction.	1.081495599	8.179630705	151.627413	2.35E−10	7.18E−07	14.99026011
TREM1	Triggering receptor expressed on myeloid cells 1, a protein that activates immune cells.	2.88932109	7.682801241	151.8534698	2.34E−10	7.18E−07	14.77673233
S100A9	S100 calcium‐binding protein A9, a protein that plays a role in inflammation and cell migration.	2.810129455	14.73337228	151.5124568	2.36E−10	7.18E−07	14.00047543
CAP1	Cysteine‐rich secretory protein 1, a protein that plays a role in innate immunity.	1.053825206	8.781068596	144.1960547	3.03E−10	7.68E−07	14.85749266
IFITM6	Interferon‐induced transmembrane protein 6, a protein that inhibits viral entry into cells.	3.236213615	10.10896756	142.1790292	3.25E−10	7.68E−07	14.81857911
CLEC4E	C‐type lectin domain containing 4E, a protein that plays a role in immune cell signaling.	1.884265255	6.006691724	137.8306926	3.79E−10	8.33E−07	13.86714715
GRINA	Glutamate receptor, ionotropic, N‐methyl D‐aspartate 4A, a protein that plays a role in excitatory synaptic transmission.	2.457471216	8.145222356	135.0814739	4.20E−10	8.37E−07	14.45129425
HP	Haptoglobin, a protein that binds hemoglobin and helps to remove it from the blood.	1.294486937	10.3230538	132.1062633	4.69E−10	8.37E−07	14.38369549
ADAM8	A disintegrin and metallopeptidase domain 8, a protein that plays a role in cell signaling and proteolysis.	2.716954621	6.90699728	130.9470476	4.90E−10	8.37E−07	14.00338238
PRG2	Proteoglycan 2, a protein that plays a role in cell–cell adhesion and extracellular matrix organization.	−1.110467479	12.80197659	−133.9604048	4.38E−10	8.37E−07	13.83421733
NGP	Neutrophil gelatinase‐associated lipocalin, a protein that plays a role in inflammation and innate immunity.	2.607431146	13.06346666	129.0539879	5.27E−10	8.53E−07	13.65562671
ANXA1	Annexin A1, a protein that plays a role in inflammation, cell death, and membrane repair.	2.113767211	10.00480998	127.2266206	5.66E−10	8.70E−07	14.23031379

### 3.2 GO and KEGG Functional Enrichment Analysis of the DEGs

**FIGURE 2:**
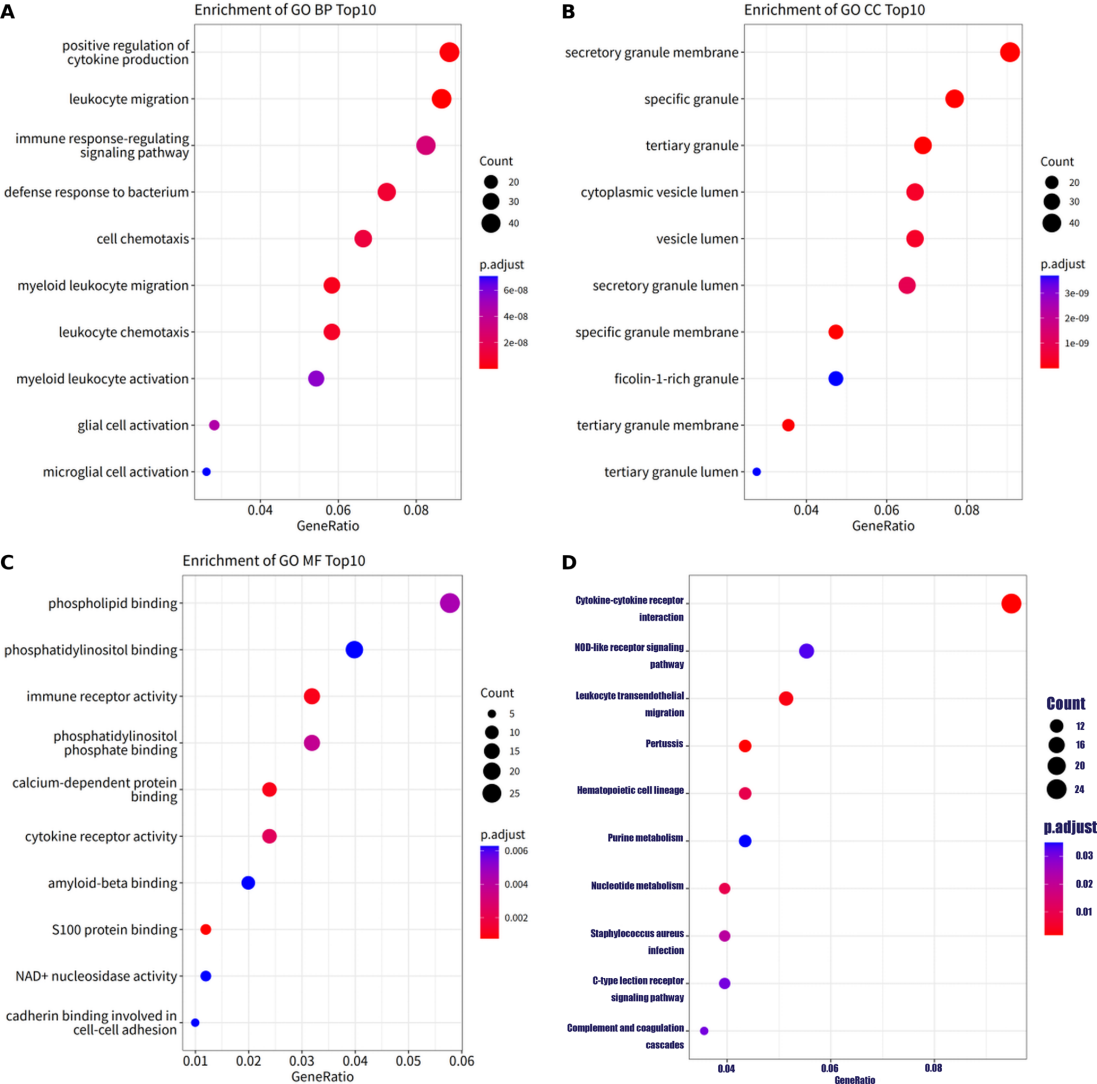
Gene ontology and KEGG pathway enrichment analysis of differentially expressed genes (DEGs) with a significance threshold of *p* < 0.05. (A) Enrichment of biological processes (GO‐BP). (B) Enrichment of cellular components (GO‐CC). (C) Enrichment of molecular functions (GO‐MF). (D) Enrichment of KEGG pathways.

This analysis led to the identification of 550 significant GO‐BP pathways, as depicted in Figure 2A. Among the top 10 enriched GO‐BP pathways, noteworthy ones include leukocyte migration, positive regulation of cytokine production, myeloid leukocyte migration, leukocyte chemotaxis, defense response to bacterium, cell chemotaxis, immune response‐regulating signaling pathway, glial cell activation, myeloid leukocyte activation, and microglial cell activation. For instance, the GO term “leukocyte migration” exhibited substantial enrichment, with differential expression observed in 43 out of 497 genes within this pathway. This finding underscores a robust association between leukocyte migration and variations in MHC‐II expression levels among HSCs. Similarly, the pathway of “positive regulation of cytokine production” was significantly enriched, highlighting the impact of MHC‐II expression levels on signaling pathways related to cytokines. Other enriched pathways such as “myeloid leukocyte migration” and “leukocyte chemotaxis” further emphasize the potential regulatory mechanisms influenced by MHC‐II levels that affect the movement of leukocytes and immune response. Additionally, the pathway of “defense response to bacterium” suggests a differential capacity in stem cells to respond to bacteria based on their expression of MHC‐II.

This approach also led to the identification of 61 significant GO‐CC pathways shown as Figure 2B. The top 10 enriched GO‐CC pathways revealed by this analysis include specific granule, tertiary granule, secretory granule membrane, specific granule membrane, tertiary granule membrane, cytoplasmic vesicle lumen, vesicle lumen, secretory granule lumen, tertiary granule lumen, and ficolin‐1‐rich granule. For example, the pathway of “specific granule” was highly enriched, showcasing significant involvement with 39 out of 507 genes being differentially expressed. This highlights the distinct cellular component changes associated with MHC‐II expression levels in HSCs, indicating a strong link between specific granule components and the cell's immunological function. Similarly, the “tertiary granule” pathway showed substantial enrichment, underlining the role of tertiary granules in the cellular activities of HSCs differing in MHC‐II expression. This reflects the intricate relationship between granule contents and immune response mechanisms. The enrichment in “secretory granule membrane” and related components further indicates that the membrane structures associated with secretory activities are significantly altered between the two stem cell states. These results suggest that the secretory apparatus of these cells, which plays a crucial role in the immune response and cell signaling, may be influenced by MHC‐II levels.

The study also identified 24 significant GO‐MF pathways shown in Figure 2C. The top 10 enriched GO‐MF pathways emerging from this analysis include S100 protein binding, immune receptor activity, calcium‐dependent protein binding, cytokine receptor activity, phosphatidylinositol phosphate binding, phospholipid binding, phosphatidylinositol binding, amyloid‐beta binding, NAD^+^ nucleosidase activity, and cadherin binding involved in cell–cell adhesion. For instance, the enrichment of the “S100 protein binding” function signifies a notable interaction between S100 protein family members and various cellular proteins, reflecting the complex regulatory mechanisms influenced by MHC‐II expression levels in HSCs. Similarly, the “immune receptor activity” pathway emphasizes the significant role of diverse receptors, including cytokine and chemokine receptors, in mediating immune responses, which appear to be modulated by the expression levels of MHC‐II in these cells. Additionally, the pathways of “calcium‐dependent protein binding” and “cytokine receptor activity” underscore the importance of calcium in cellular signaling and the role of cytokines in the immune response, both of which are influenced by MHC‐II expression. The pathways related to “phosphatidylinositol phosphate binding” and “phospholipid binding” suggest alterations in lipid‐mediated signaling processes associated with MHC‐II levels in HSCs.

This analysis was conducted using a stringent significance threshold of *p*.adjust < 0.05, leading to the identification of 16 significant KEGG pathways shown as Figure 2D and Table 2. The top 10 enriched KEGG pathways from this analysis include Cytokine‐cytokine receptor interaction, Pertussis, Leukocyte transendothelial migration, Hematopoietic cell lineage, Nucleotide metabolism, *Staphylococcus aureus* infection, Complement and coagulation cascades, C‐type lectin receptor signaling pathway, NOD‐like receptor signaling pathway, and Purine metabolism. For example, the “Cytokine‐cytokine receptor interaction” pathway showed significant enrichment, indicating a robust interplay between cytokines and their receptors, which influences the behavior of HSCs based on their MHC‐II expression levels. This pathway involved 24 DEGs, underscoring the complexity and significance of cytokine signaling in these cells. Similarly, the “Pertussis” and “Leukocyte transendothelial migration” pathways exhibited significant enrichment. The former suggests a potential susceptibility or response mechanism of the cells to bacterial infections, while the latter emphasizes the importance of leukocyte movement across endothelial barriers, which could be influenced by the MHC‐II status. The “Hematopoietic cell lineage” pathway underscores the differentiation processes of HSCs and their specification into specific lineages, which seem to be significantly different between MHC‐II‐high and MHC‐II‐low cells. Meanwhile, the pathways of “Nucleotide metabolism” and “Purine metabolism” demonstrate significant alterations in metabolic processes associated with nucleotides, which are essential for cell division and DNA/RNA synthesis, exhibiting variations based on MHC‐II expression. Infection‐related pathways such as “ *Staphylococcus aureus* infection,” “Complement and coagulation cascades,” and “C‐type lectin receptor signaling pathway” exhibit notable enrichment. These findings highlight the potential differences in immune response and bacterial interaction mechanisms between the two stem cell groups. Finally, the enrichment of the “NOD‐like receptor signaling pathway” suggests involvement of intracellular detection mechanisms for stress or pathogens that may play distinct roles in MHC‐II‐high vs. MHC‐II‐low HSCs.

**TABLE 2:** Top KEGG pathways significantly enriched among the DEGs.

ID	Description	GeneRatio	BgRatio	*p*	*p*.adjust	*q*	geneID	Count
hsa04060	Cytokine‐cytokine receptor interaction	24/253	297/8662	5.67E−06	0.001569815	0.00137206	3579/1439/8807/1441/1230/7850/3553/94/3572/163702/3624/6348/4352/1524/657/3597/3569/3557/3577/4050/8742/8744/9573/5473	24
hsa05133	Pertussis	11/253	76/8662	1.13E−05	0.001569815	0.00137206	3684/3689/3553/3394/5603/114609/4843/3569/714/721/712	11
hsa04670	Leukocyte transendothelial migration	13/253	115/8662	2.85E−05	0.002629656	0.002298389	4318/3684/3689/653361/60/87/5579/7408/5603/50848/4689/9076/4633	13
hsa04640	Hematopoietic cell lineage	11/253	99/8662	0.000137939	0.009546471	0.008343866	3684/945/1441/7850/3553/928/3672/3569/952/1604/924	11
hsa01232	Nucleotide metabolism	10/253	85/8662	0.000172319	0.009546471	0.008343866	9615/7498/272/953/956/7378/4907/158067/100/4832	10
hsa05150	*Staphylococcus aureus* infection	10/253	96/8662	0.000469012	0.021652719	0.018925045	820/728/3684/3689/2358/2357/714/5724/721/712	10
hsa04610	Complement and coagulation cascades	9/253	86/8662	0.00086715	0.030644656	0.026784233	728/3684/3689/5329/1604/714/721/1191/712	9
hsa04625	C‐type lectin receptor signaling pathway	10/253	104/8662	0.000885044	0.030644656	0.026784233	26 253/3553/3709/1263/5603/338339/3569/1960/602/6237	10
hsa04621	NOD‐like receptor signaling pathway	14/253	186/8662	0.001075671	0.033106778	0.028936192	820/7226/3553/3709/4940/5027/91662/5603/118429/79671/4939/3569/598/6041	14
hsa00230	Purine metabolism	11/253	128/8662	0.001265516	0.035054802	0.030638816	9615/7498/272/953/5153/956/4907/158067/100/5138/4832	11
hsa05323	Rheumatoid arthritis	9/253	93/8662	0.001519685	0.038268424	0.033447606	3689/3553/6348/1513/535/3569/4050/245972/1493	9
hsa05140	Leishmaniasis	8/253	77/8662	0.001749525	0.040384865	0.035297431	3684/3689/3553/653361/5579/5603/4689/4843	8
hsa00561	Glycerolipid metabolism	7/253	63/8662	0.002287979	0.046066395	0.040263237	9388/8694/2710/84649/160851/10555/84803	7
hsa05152	Tuberculosis	13/253	180/8662	0.002328265	0.046066395	0.040263237	26 253/820/3684/3689/3553/1263/5603/114609/4843/535/3569/10333/245972	13
hsa04061	Viral protein interaction with cytokine and cytokine receptor	9/253	100/8662	0.002519119	0.046519733	0.040659467	3579/8807/1230/3572/6348/1524/3569/3577/5473	9
hsa05146	Amoebiasis	9/253	102/8662	0.002884187	0.04993249	0.043642306	3684/3689/7850/3553/87/5579/384/4843/3569	9

**FIGURE 3:**
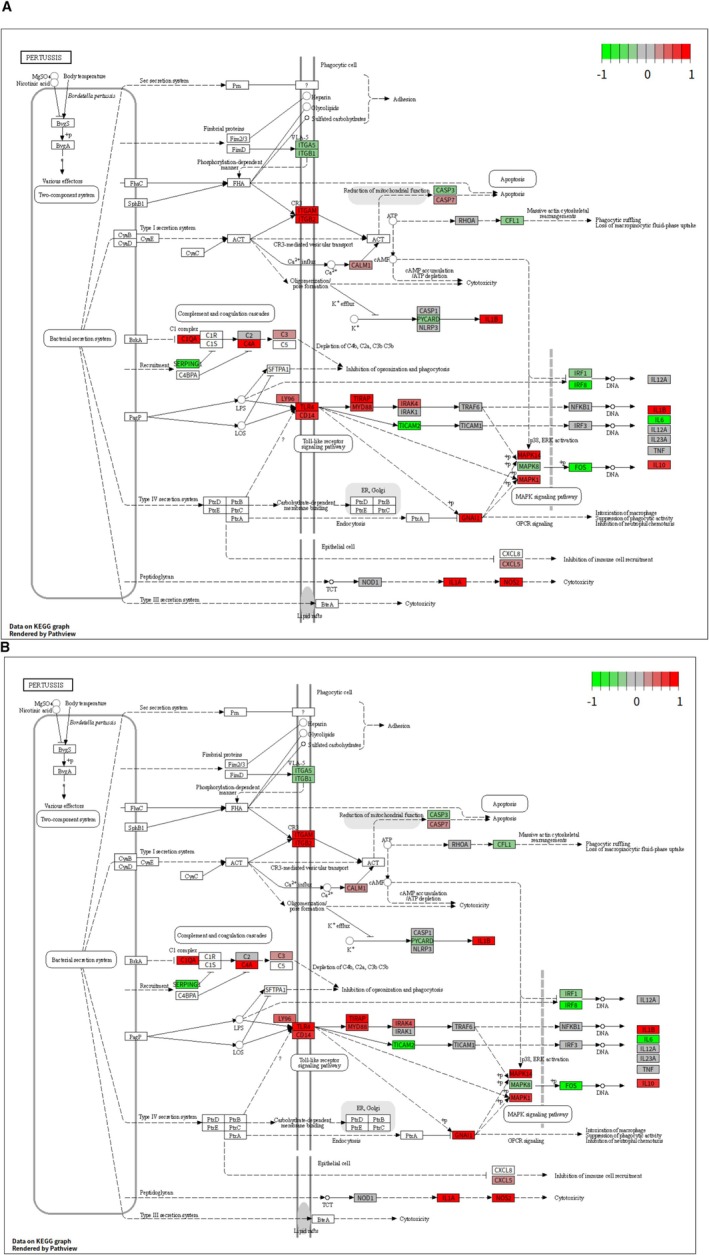
Top two pathway visualizations with LogFC. (A) hsa04060 with LogFC. (B) hsa05133 with LogFC.

The pathway diagrams were created for the following two KEGG pathways with the R package Pathview shown as Figure 3:

– hsa04060 (Cytokine‐cytokine receptor interaction): Diagrams (Figure 3A) illustrate this pathway, with the logFC. This pathway is central to the communication between cells of the immune system through cytokines and their receptors, playing a critical role in initiating and modulating immune responses.
– hsa05133 (Pertussis): Diagrams (Figure 3B) visualize this pathway. Pertussis, or whooping cough, is caused by the bacterium *Bordetella pertussis* , and the pathway illustrates the host immune response to this infection, providing insights into how different gene expression levels can influence this response.

### 3.3 Exploring Biological Pathways: Insights From Reactome Enrichment Analysis

**FIGURE 4:**
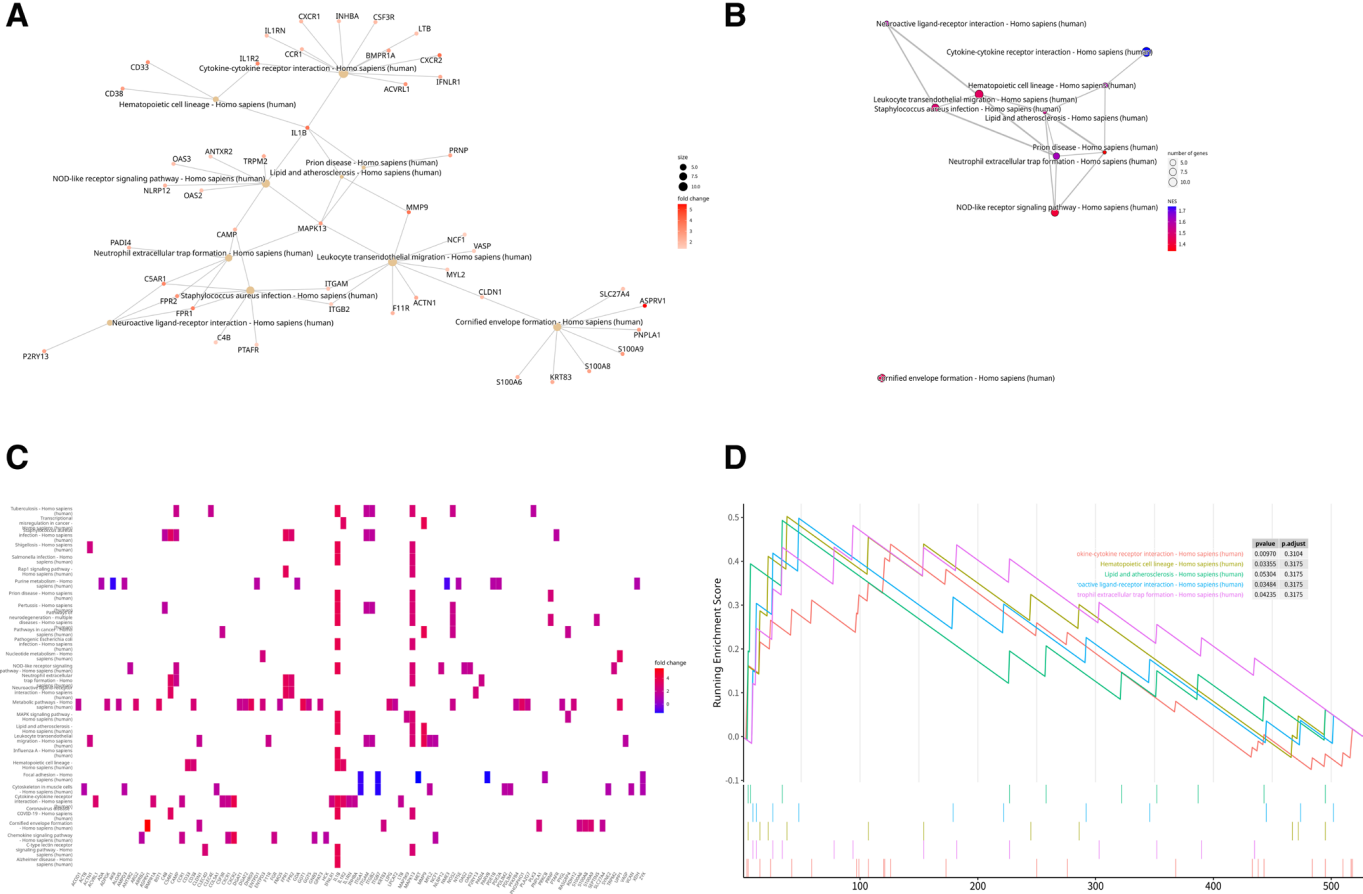
Pathway interaction and activity analysis in HSC subgroups. (A) Cnetplot: Network analysis of pathway interactions. This network plot illustrates the interactions and connections between different pathways. (B) Emapplot: Visualizing pathway crossovers. This emap plot highlights the crossovers and overlaps between pathways, indicating shared genes and functional relationships. (C) Heatplot: Pathway activity heatmap in HSC subgroups. This heatmap shows the activity levels of different pathways across HSC subgroups, with colors representing the degree of enrichment. (D) Gene set enrichment analysis (GSEA) results visualization: Gseaplot. This gseaplot visualizes the results of GSEA, showing the enrichment scores and leading edge subsets for significant pathways.

The Reactome enrichment analysis used a significance threshold of *p*.adjust < 0.05; it is noteworthy that no pathways exhibited significant changes under this threshold. Nevertheless, for illustrative purposes, the top 10 pathways (Neutrophil Degranulation, Innate Immune System, Immune System, Adaptive Immune System, Extracellular Matrix Organization, Signal Transduction, Class A/1 [Rhodopsin‐like receptors], GPCR Ligand Binding, G alpha (i) Signaling Events, and Transport of Small Molecules) identified by the analysis are presented despite not surpassing the adjusted *p* value threshold. Each of these pathways, as identified in the analysis, contributes to understanding the biological processes and signaling mechanisms that are potentially differentiated between MHC‐II‐high and MHC‐II‐low HSCs. While none met the stringent threshold for significance in this particular analysis, they represent areas of interest for further investigation. The visual representations provided through cnetplot (Figure 4A), emapplot (Figure 4B), heatplot (Figure 4C), and gseaplot (Figure 4D) offer a structured view into these complex biological interactions and processes.

### 3.4 Investigating Genetic Links: MR Analysis for Breast Cancer

In this MR study, we utilized SNPs associated with DEGs between MHC‐II‐high and MHC‐II‐low HSCs as instrumental variables to investigate their potential causal relationship with breast cancer risk. A total of 268 SNPs were analyzed using several MR methods, including MR Egger, weighted median, IVW, simple mode, and weighted mode.

**FIGURE 5:**
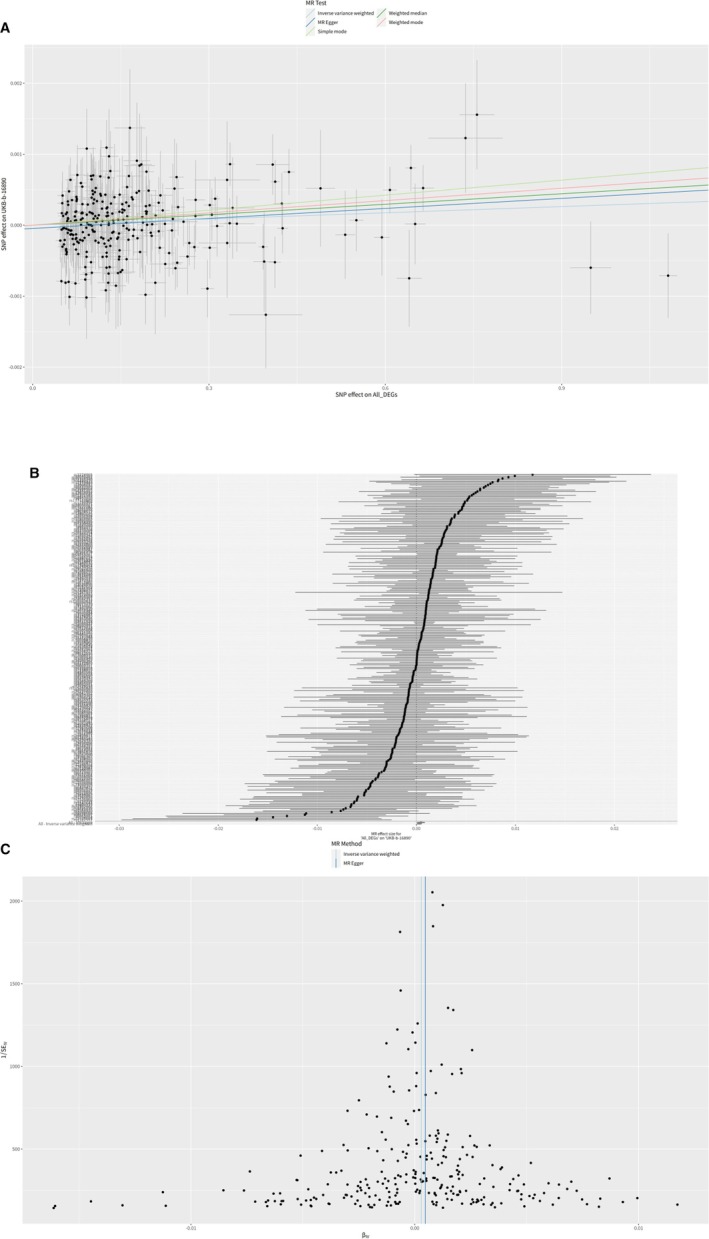
MR analysis for breast cancer. (A) Scatter plot. Association between genetic variants and breast cancer risk. (B) Forest plot. Effect sizes and confidence intervals for the association between genetic variants and breast cancer risk. (C) Funnel plot. Assessment of publication bias in the MR analysis.

The results, depicted in a scatter plot (Figure 5A), demonstrate a consistent trend where the effects of SNPs on DEGs appear to marginally elevate the susceptibility to breast cancer. This trend is illustrated by diverse lines for each MR method, indicating a potential association. Specifically, the MR Egger and weighted median methods suggest a small yet positive association between the DEGs and breast cancer, with *β* coefficients approximately around 0.0005 and *p* values of 0.0150 and 0.0202, respectively. The IVW method indicates a weaker association with a *β* of 0.0003 and a *p* value of 0.0178, while the simple mode method, despite having a higher *β* coefficient of 0.0007, exhibits a higher *p* value of 0.1293, indicating reduced reliability. On the other hand, the weighted mode method demonstrates a stronger link with a *β* coefficient of 0.0006 and the most significant *p* value of 0.0110.

The forest plot (Figure 5B) aggregates the estimates from different MR analyses, showing point estimates and 95% confidence intervals for each method. This visualization underscores the varied strengths of association detected by different MR methods, with weighted mode showing the strongest association between DEGs and breast cancer risk, highlighted by the lowest *p* value among the methods. Additionally, heterogeneity tests indicated no significant disparity among the SNPs used for the MR analyses (*p* > 0.27 for both MR Egger and IVW methods), suggesting a consistent impact of the instrumental variables across the different methodologies. The funnel plot (Figure 5C) was constructed to evaluate potential bias and demonstrates a distribution that exhibits relative symmetry around the zero‐effect line, consistent with the findings from Egger's intercept indicating no significant directional pleiotropy (*p* = 0.227). This symmetrical pattern provides support for the credibility of the SNP selection and suggests a minimal likelihood of bias influencing the MR analysis.

**FIGURE 6:**
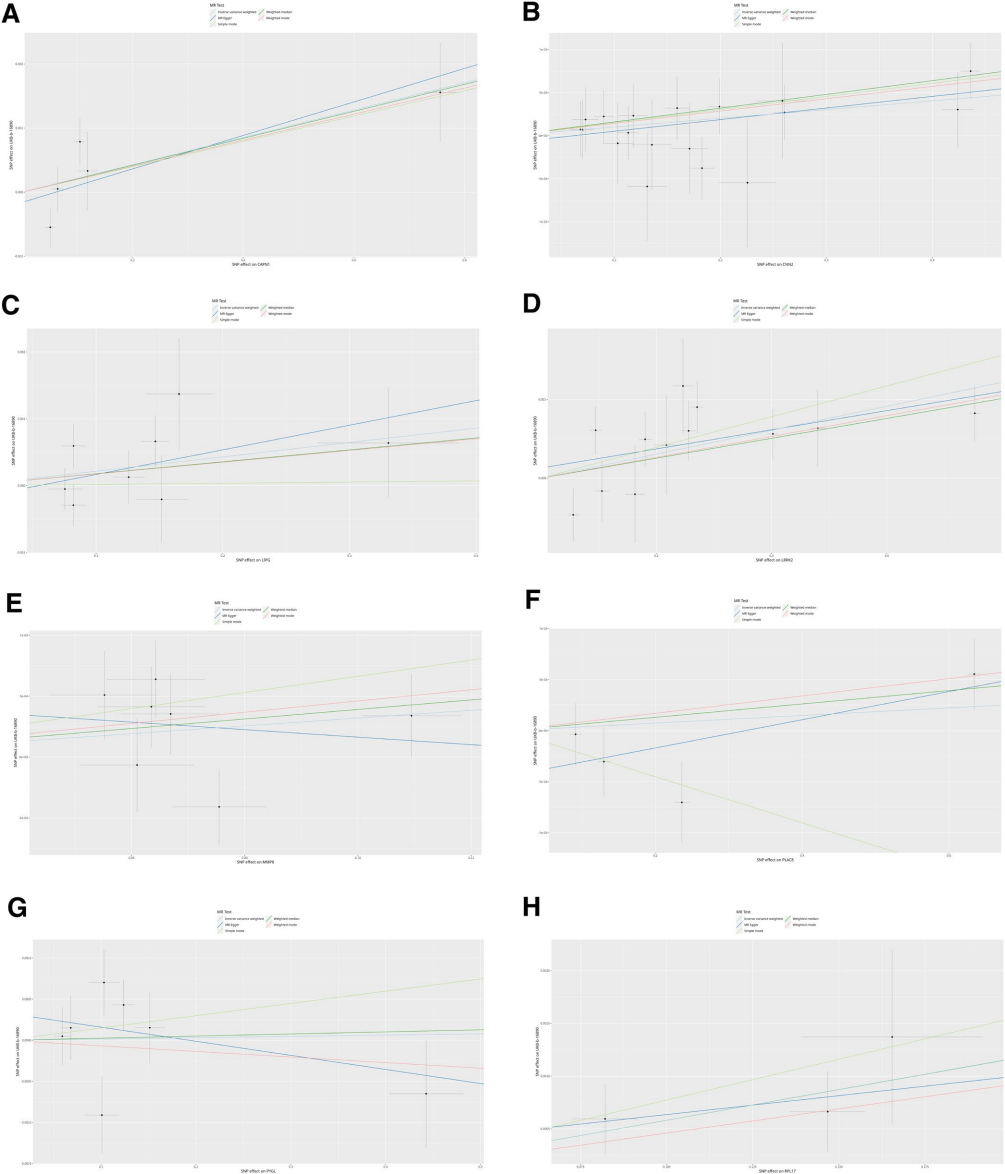
Correlation with breast cancer susceptibility, scatter plot, forest plots, and funnel plots of MR analysis for the DEGs. (A) CAPN1. (B) CNN2. (C) LIPG. (D) LRRK2. (E) MMP8. (F) PLAC8. (G) PYGL. (H) RPL17.

In this comprehensive analysis shown as Figure 6, each SNP associated with DEGs between MHC‐II‐high and MHC‐II‐low HSCs was individually investigated as an instrumental variable to evaluate its association with breast cancer risk. Only a limited number of DEGs were found to possess SNPs that exhibit a potential relationship with breast cancer risk.

– CAPN1: This gene's associated SNPs showed varied results across different MR methods: MR Egger did not show a significant association (*b* = 0.0019, *p* = 0.4448). Weighted Median and IVW methods suggested a small positive association with breast cancer risk (*b* = 0.0023 and *b* = 0.0024, respectively), with *p* values indicating potential significance (*p* = 0.0228 and *p* = 0.0190, respectively). Simple mode and weighted mode analyses were not significant.
– CNN2: Analysis of CNN2‐related SNPs also provided mixed outcomes: No significant association was found using MR Egger (*b* = 0.0015, *p* = 0.1267). Weighted median, IVW, and weighted mode methods indicated a weaker association (lowest *p* = 0.0475 for weighted mode).
– LIPG: For this gene, MR Egger analysis showed no significant relationship (*b* = 0.0003, *p* = 0.9321). However, the IVW method indicated a small positive association with breast cancer risk (*b* = 0.0032, *p* = 0.0272).
– MMP8: The results were again mixed with no significant findings from MR Egger and simple mode methods. A potential association from the IVW method (*b* = 0.0044, *p* = 0.0444).
– PLAC8 and RPL17: Both showed no significant results in MR Egger, but some evidence of a possible association in the weighted median and IVW methods, especially for RPL17 with a more substantial *β* value in weighted median analysis (*b* = 0.0073, *p* = 0.0266).

### 3.5 Investigating Genetic Links: PD‐L1 Expression Levels

**FIGURE 7:**
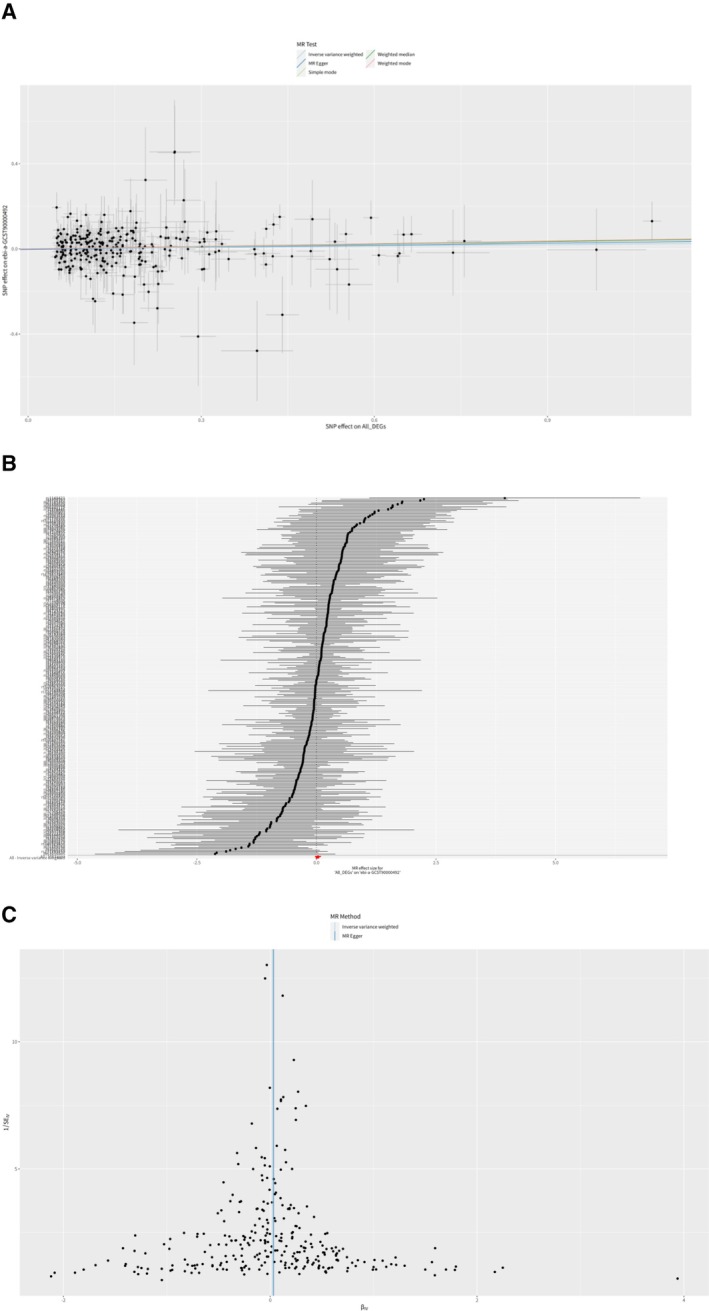
MR analysis for PD‐L1 status. (A) Scatter plot. Association between genetic variants and PD‐L1 status. Each point represents a genetic variant, with the *x*‐axis showing the effect size (e.g., log odds ratio) and the *y*‐axis showing the standard error. (B) Forest plot. Effect sizes and 95% confidence intervals for the association between genetic variants and PD‐L1 status. Each row represents a genetic variant, with the effect size and confidence interval displayed. (C) Funnel plot. Assessment of publication bias in the MR analysis. Symmetry of the points around the center line indicates low publication bias.

The focus of this MR analysis was to investigate the causal relationship between PD‐L1 expression levels, which are influenced by MHC‐II. Our analysis, as shown in Figure 7, primarily utilized instrumental variables derived from SNPs associated with DEGs that are linked to MHC‐II levels in the data set ebi‐a‐GCST90000492. The MR Egger analysis revealed no significant heterogeneity among the instrumental variables (*Q* = 293.533, df = 280, *p* = 0.2770), indicating that the genetic instruments employed exhibited a relatively homogeneous distribution across the analyzed SNPs. The findings were further supported by the IVW method, which also indicated no significant heterogeneity (*Q* = 293.7320, df = 281, *p* = 0.2890), aligning with the critical assumption of homogeneity necessary for a valid MR analysis. Despite the genetic instruments exhibiting homogeneity, there was no substantial evidence of directional pleiotropy according to Egger's intercept (intercept = −0.0027, SE = 0.0061, *p* = 0.6630). This reinforces the assumption that the SNPs used could serve as reliable instruments for exploring the relationship between PD‐L1 levels within this MR framework. Nevertheless, causal estimates obtained from various MR methods (MR Egger, weighted median, and IVW) did not reveal any statistically significant associations between PD‐L1 expression levels. Specifically, the MR Egger method yielded an effect estimate of *b* = 0.03266 with a standard error of SE = 0.03166 (*p* = 0.303). Similarly, the Weighted Median method provided an effect estimate of *b* = 0.0381 with SE = 0.0334 (*p* = 0.2530), while the IVW approach reported a lower effect estimate of *b* = 0.0220 with SE = 0.0199 (*p* = 0.2710). In all instances, the *p* values did not reach the conventional level of statistical significance.

**FIGURE 8:**
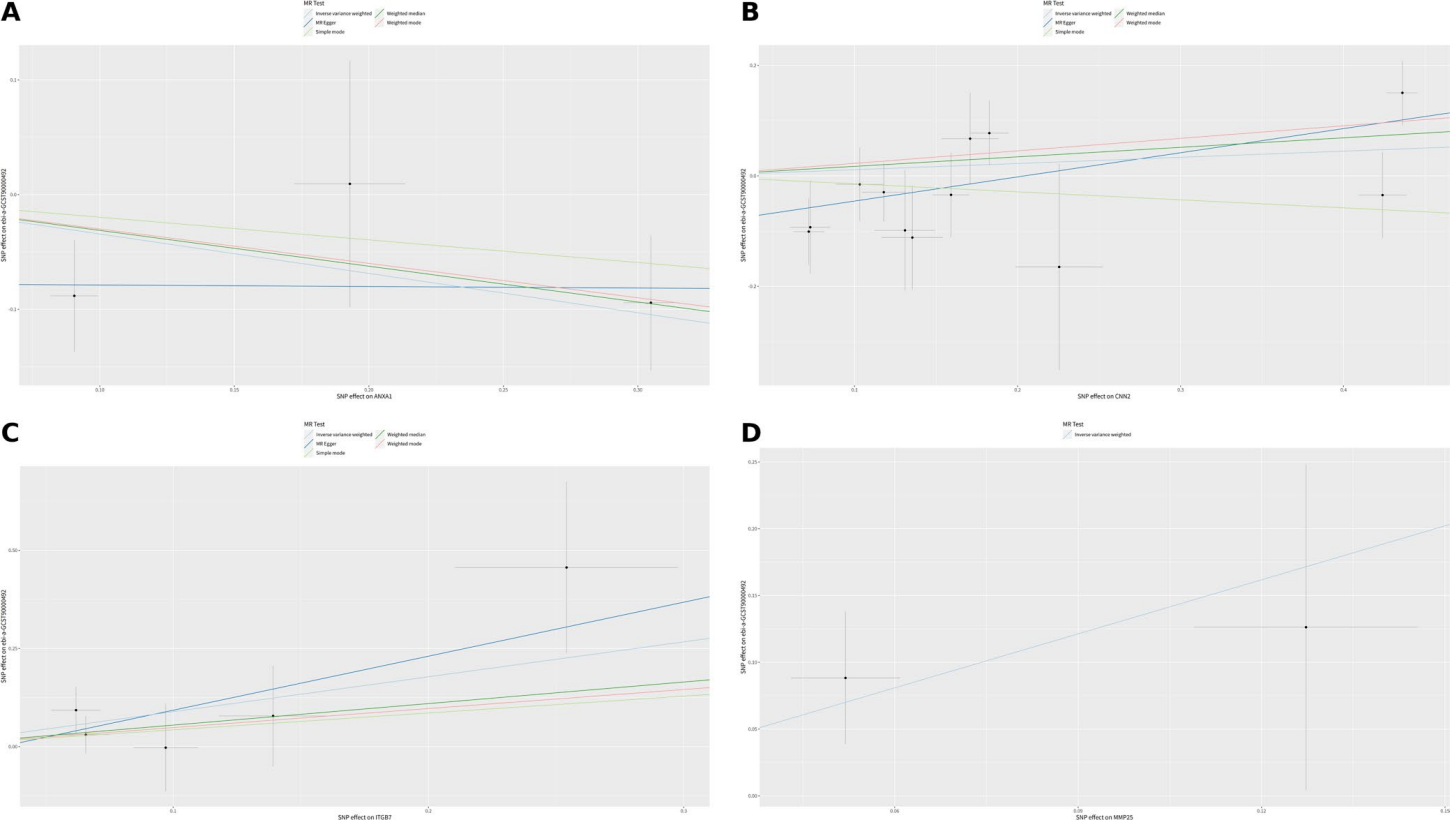
Correlation with PD‐L1 status: Scatter plot, forest plots, and funnel plots of MR analysis for the DEGs. (A) ANXA1. (B) CNN2. (C) ITGB7. (D) MMP25.

In our study, although the MR analysis using all SNPs associated with DEGs between MHC‐II‐high and MHC‐II‐low groups did not yield statistically significant associations, individual analyses of each DEG related to MHC‐II revealed specific relationships with the PD‐L1 status as depicted in Figure 8. The DEG ANXA1 exhibited a significant inverse relationship with PD‐L1 status, as evidenced by two separate analyses using the IVW method with three SNPs each. The effect sizes (*b*) were −0.3435, and the standard errors (SEs) were 0.1724, resulting in *p* values of 0.0463, indicating a significant inverse relationship. In the case of the gene CNN2, an analysis performed using the MR Egger method with 12 SNPs indicated a positive association with PD‐L1 status, evidenced by an effect size of 0.4375, a standard error of 0.1642, and a *p* value of 0.0237, suggesting a significant relationship. The IVW method with five SNPs revealed a robust positive association between ITGB7 and PD‐L1 status, demonstrating an effect size of 0.8894 (SE = 0.3981, *p* = 0.0255), thereby emphasizing another significant correlation. The DEG MMP25, analyzed through the IVW method with two SNPs, also showed a positive and significant association with PD‐L1 status, demonstrated by an effect size of 1.3480, a standard error of 0.6763, and a *p* value of 0.0462. Finally, the gene PTMA, analyzed using the IVW method with two SNPs, also exhibited a significant positive association with PD‐L1 status (effect size = 1.3480, standard error = 0.6763, *p* value = 0.0462).

### 3.6 Investigating CAPN1 in Breast Cancer Progression

**FIGURE 9:**
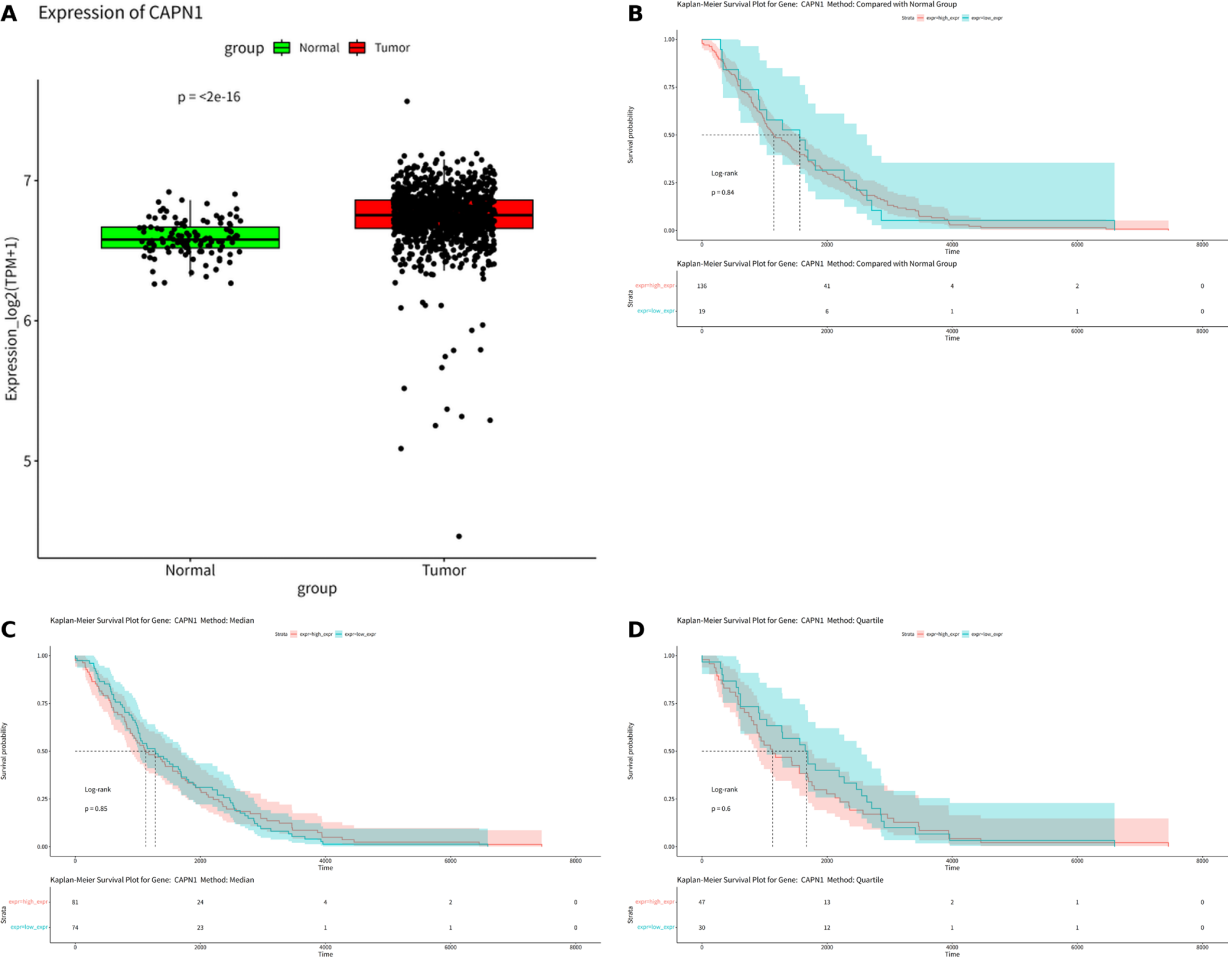
CAPN1 expression and its impact on breast cancer survival. (A) CAPN1 boxplot. (B) KM survival curve using Algorithm 1 (compared to normal). (C) KM survival curve using Algorithm 2 (median). (D) KM survival curve using Algorithm 3 (quartiles).

**FIGURE 10:**
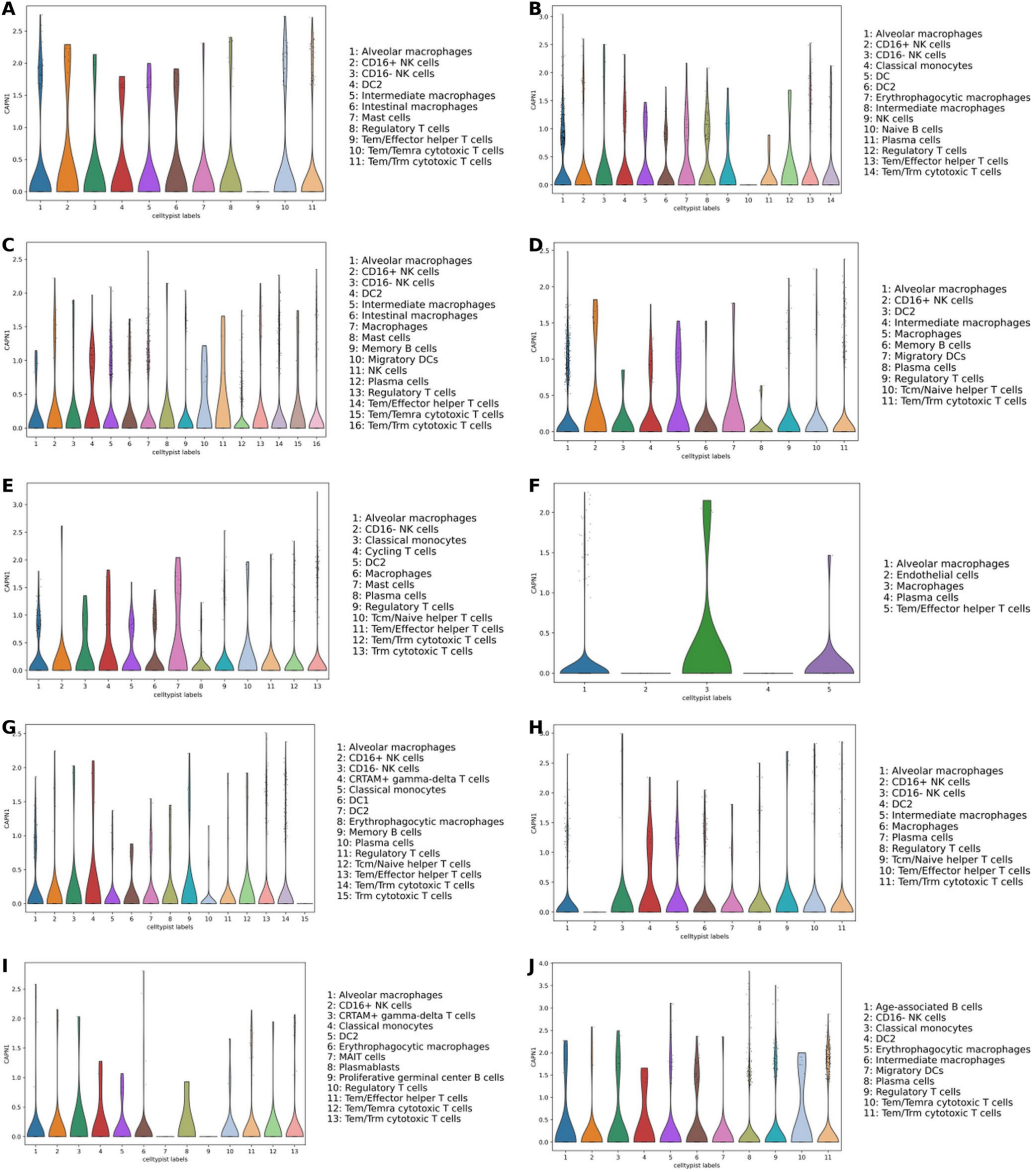
Distribution of CAPN1 expression in TNBC immune cells. (A) h06B_P. (B) h57B_P. (C) h10B_P. (D) h15B_P. (E) h17B_P. (F) h26B_P. (G) h39B_P. (H) h45B_P. (I) h52B_P. (J) h53B_P.

In our study, MR on breast cancer confirmed that among the genes associated with high and low MHC‐II expression. Specifically, we identified nine genes (CAPN1, CNN2, LIPG, LRRK2, MMP8, PLAC8, PYGL, RPL17, and SYNE1) that showed significant associations. Notably, CAPN1 has been extensively investigated in previous literature and is suggested to be potentially associated with the incidence and prognosis of breast cancer. Our research specifically focused on examining the expression of CAPN1 in breast cancer. Our findings demonstrate a significant differential expression of CAPN1 between normal and cancerous breast tissues, with a *p* value < 2e−16 (Figure 9A). Furthermore, we performed survival analysis using Kaplan–Meier curves based on the TCGA‐BRCA data set to compare the relationship between CAPN1 expression levels and survival rates through three different algorithms: (1) compared with normal tissues (Figure 9B), (2) median (Figure 9C), and (3) quartiles (Figure 9D). Intriguingly, these analyses did not reveal a significant correlation between CAPN1 expression levels and breast cancer survival rates. Additionally, our analysis of single‐cell sequencing data from 11 groups of TNBC patients resistant to PD‐1 inhibitors (who did not achieve pCR) revealed a significant expression of CAPN1 across diverse immune cell populations in the immune cell data set (Figure 10). This finding suggests that CAPN1 may play a role in the immune response associated with TNBC and resistance to PD‐1 inhibitors.

### 3.7 Investigating ANXA1 With PD‐L1 Status

**FIGURE 11:**
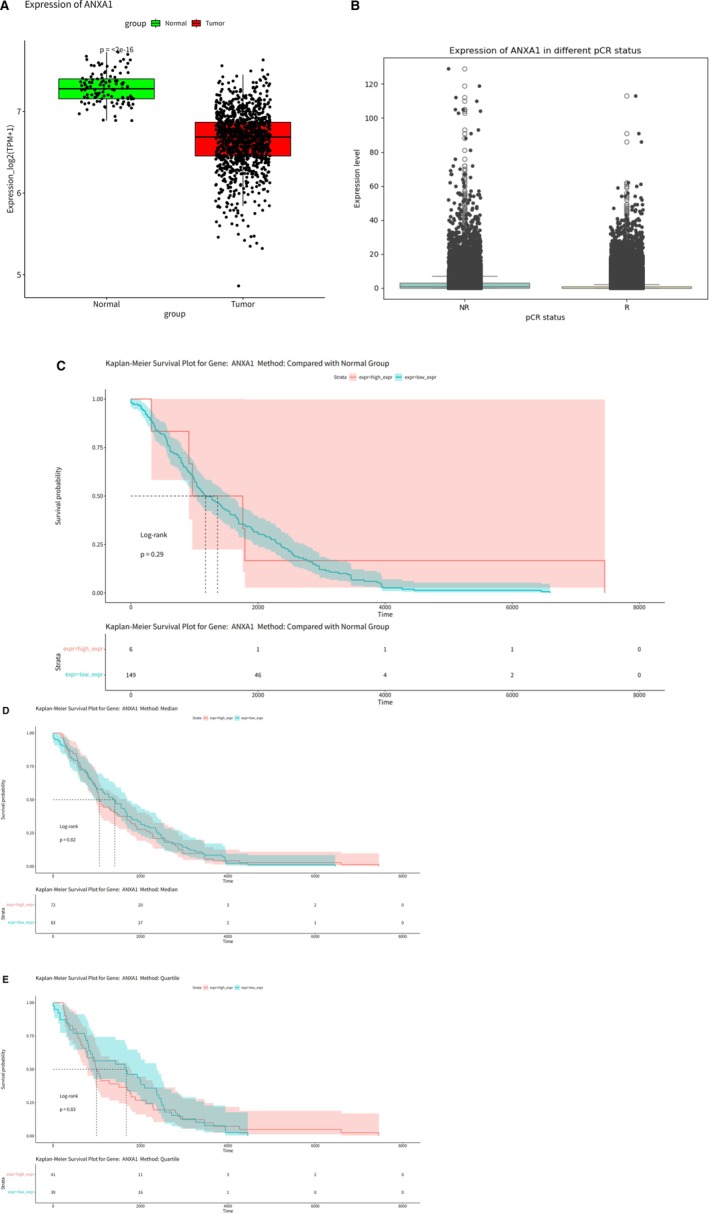
ANXA1 expression and its impact on PD‐L1 status. (A) ANXA1 expression boxplot. (B) ANXA1 expression in different pCR states. (C) KM survival curve using Algorithm 1 (compared to normal). (D) KM survival curve using Algorithm 2 (median). (E) KM survival curve using Algorithm 3 (quartiles).

**FIGURE 12:**
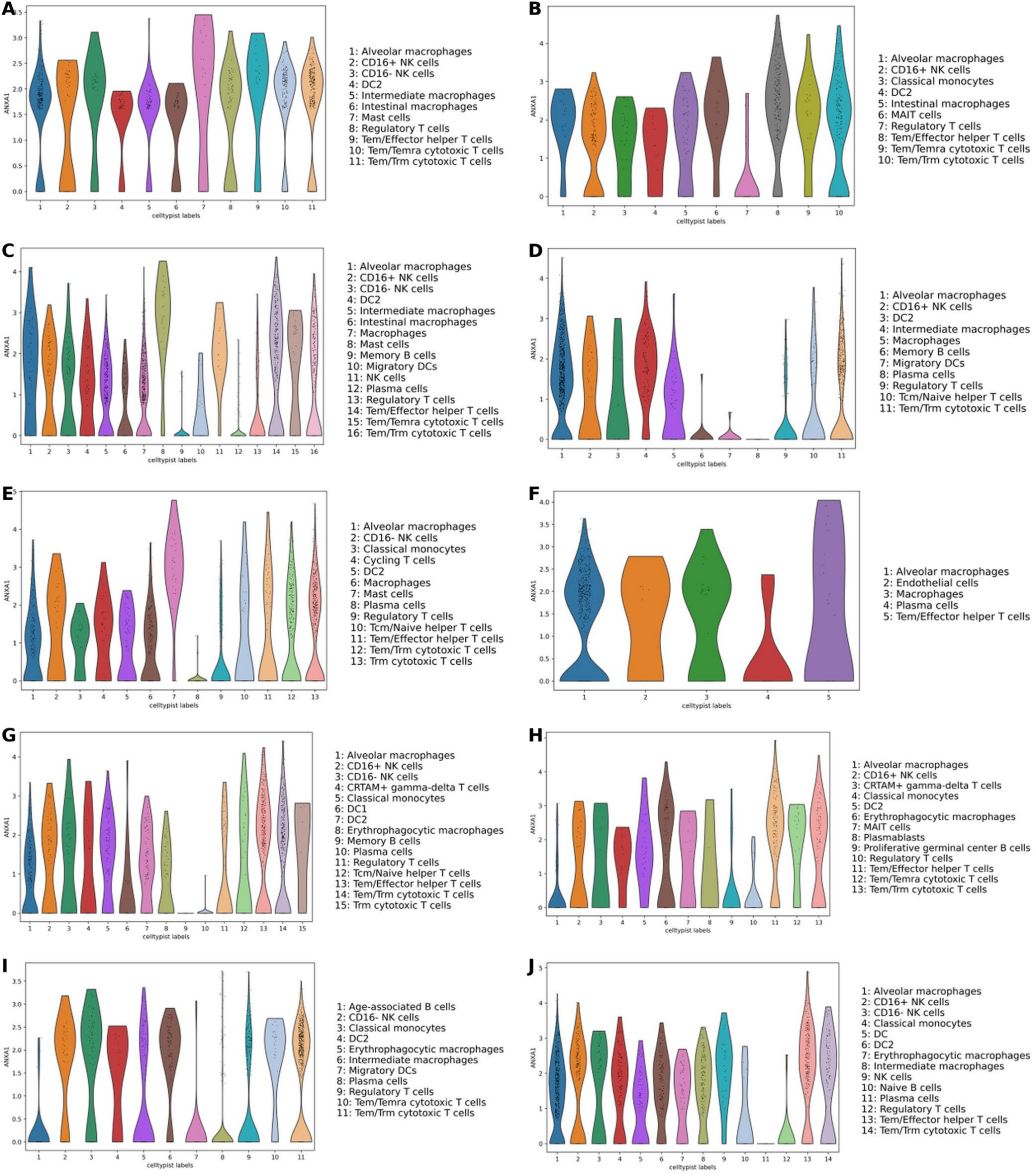
Distribution of ANXA1 expression in TNBC immune cells. (A) h06B_P. (B) h64B_P. (C) h10B_P. (D) h15B_P. (E) h17B_P. (F) h26B_P. (G) h39B_P. (H) h45B_P. (I) h52B_P. (J) h53B_P.

Among the five PD‐L1 status‐associated genes obtained from Mendel's randomized analysis in MHC‐II related genes (ANXA1, CNN2, ITGB7, MMP25, and PTMA), ANXA1 has been widely reported to be potentially associated with PD‐1/PD‐L1 resistance; therefore, we focused on analyzing the association of ANXA1 with breast cancer and its association with PD‐1/PD‐L1 resistance. Our analysis of the TCGA‐BRCA breast cancer database revealed a significant differential expression of ANXA1 between normal and cancerous breast tissues, with ANXA1 being notably downregulated in cancer tissues (*p* < 2e−16) (Figure 11A). The mean expression level of ANXA1 in single‐cell sequencing data is higher in the NR (nonresponder) group compared to the R (responder) group, indicating that the expression of ANXA1 is elevated in patients who did not achieve a pathologic complete response (Figure 11B). Survival analyses conducted using the TCGA‐BRCA data set, employing three different algorithms: Compared with Normal (Figure 11C), Median (Figure 11D), and Quartiles (Figure 11E), did not show a significant correlation between ANXA1 expression levels and breast cancer survival rates. Our examination of single‐cell sequencing data from 11 groups of TNBC patients, who were resistant to PD‐1/PD‐L1 drugs and did not achieve pCR, revealed that ANXA1 was significantly expressed across various types of immune cells within the immune cell data set (Figure 12). This highlights ANXA1's involvement in the immune landscape of TNBC, particularly in the context of PD‐1/PD‐L1 drug resistance.

## 4 Discussion

The enrichment in cellular components related to secretory and granule membranes, as indicated by our GO‐CC pathway results, aligns with findings that suggest alterations in immune cell granulogenesis and secretion contribute to tumor cells evading immune surveillance [20].

Disrupted secretory mechanisms, potentially resulting in reduced antigen presentation and cytokine signaling, may contribute to the lack of response to PD‐1/PD‐L1 therapies.

In our comprehensive study, MR analysis confirmed that nine genes (CAPN1, CNN2, LIPG, LRRK2, MMP8, PLAC8, PYGL, RPL17, and SYNE1) associated with high and low MHC‐II expression are potentially closely linked to breast cancer. Specifically, recent literature has highlighted the significant association of CAPN1 with breast cancer and particularly TNBC. This finding further strengthens its potential as a biomarker and therapeutic target [21, 22, 23, 24, 25]. Our bioinformatics analyses, utilizing data from the TCGA‐BRCA breast cancer database encompassing a diverse range of breast cancer samples and corresponding normal tissues, have unveiled significant differential expression of CAPN1 between normal and cancerous tissues, thereby emphasizing its potential involvement in oncogenic mechanisms. However, this pronounced differential expression does not correlate significantly with breast cancer prognosis, suggesting that while CAPN1 may be involved in the etiology or progression of breast cancer, its direct impact on patient outcomes is not definitive. When investigating CAPN1's role in the immune response, we observed that CAPN1 expression was significantly present across various immune cell populations in single‐cell sequencing data from TNBC patients resistant to PD‐1 inhibitors. This finding suggests that CAPN1 might influence immune modulation in the TME and could play a role in the resistance mechanisms observed in patients who do not achieve pCR to PD‐1 inhibitors. In particular, the involvement of CAPN1 in the immune response aligns with its role in regulating immune cell functions, including the activation and polarization of immune cells, which may impact the effectiveness of immune checkpoint inhibitors in TNBC. This complexity underscores the need for further research to elucidate CAPN1's intricate role in breast cancer pathology and clarify its implications for future treatment strategies.

In our comprehensive analysis, ANXA1 has emerged as a significant factor affecting the efficacy of PD‐1/PD‐L1 therapies. This finding is supported by recent studies that collectively underscore the multifaceted role of ANXA1 in cancer immunotherapy and its potential as a biomarker for therapeutic outcomes. Lee et al. [26] elucidated the connection between RRM2 expression and the immune microenvironment in lung adenocarcinoma (LUAD), suggesting RRM2 as a predictive biomarker for PD‐1/PD‐L1 inhibitors, primarily through the ANXA1/AKT signaling pathway. This finding highlights the intricate network involving ANXA1 and its impact on the immunotherapeutic response, particularly in LUAD patients [26]. Furthermore, Xiao et al. [27] demonstrated that ANXA1 contributes to tumor immune evasion by influencing the interaction between PARP1 and Stat3, leading to the upregulation of PD‐L1 expression across various cancers. This mechanistic insight provides a new perspective on how ANXA1 facilitates an immunosuppressive environment, promoting cancer progression and resistance to immunotherapies [27]. Shen et al. [28] extended upon this understanding by demonstrating a correlation between the expression levels of various members within the ANXA1 family and the outcomes of anti‐PD‐1/PD‐L1 therapies across diverse cancer types. This comprehensive analysis spanning multiple cancers further underscores the significance of ANXA1 and its family members in modulating immune responses, highlighting their potential as predictive markers for immunotherapy efficacy [28]. Moreover, Yu et al. [29] have revealed that an ANXA1‐derived peptide called A11 can effectively reduce the stability of PD‐L1 through the ubiquitin‐proteasome pathway. This discovery presents a novel therapeutic strategy to combat tumor immune evasion. Their research not only provides evidence of the therapeutic potential of targeting ANXA1 but also demonstrates the synergy between A11 and PD‐1 monoclonal antibodies in enhancing antitumor effects [29]. Finally, the work by Xiong et al. (2021) on renal cancer (RCC) highlights the broader implications of RRM2 and ANXA1 beyond their traditional roles [30]. They discovered that RRM2 stabilizes ANXA1, thereby activating the AKT pathway independently of its conventional enzymatic activities. This mechanism contributes to the development of resistance to sunitinib in RCC and implicates RRM2 and ANXA1 in the modulation of resistance toward PD‐1/PD‐L1 therapy. Tumor cells may exacerbate this effect by upregulating ANXA1 and activating related signaling pathways, such as AKT, thereby establishing a negative feedback loop that impedes the efficacy of PD‐1/PD‐L1 blockade therapies. Additionally, the upregulation of ANXA1 may represent an immune regulatory mechanism aimed at attenuating inflammation and facilitating tissue repair. However, within the context of PD‐1/PD‐L1 blockade resistance, this regulatory response could potentially have adverse effects by fostering a more immunosuppressive tumor microenvironment and further compromising T cell functionality and immune surveillance. The complexity of the tumor microenvironment is highlighted by single‐cell sequencing studies showing high ANXA1 expression across all immune cells in the context of PD‐1/PD‐L1 drug resistance, suggesting an adaptive response of the tumor microenvironment to ICB treatments.

The downregulation of ANXA1 in breast cancer tissues under normal conditions may indicate the tumor's evasion of an effective immune response. However, in patients nonresponsive to PD‐1 blockade, the upregulation of ANXA1 within immune cells could potentially serve as an adaptive resistance mechanism. This paradoxical increase suggests that while the tumor initially evades immune detection through low ANXA1 levels, under the selective pressure of immunotherapy, it may upregulate ANXA1 in immune cells to enhance its immunosuppressive environment. This could create a more tolerogenic environment, hampering the reactivation of T cells targeted by PD‐1/PD‐L1 therapies and leading to therapeutic resistance. Elevated ANXA1 levels in the immune cells of PD‐1 nonresponders may also contribute to the polarization of macrophages towards an M2 phenotype, which is well‐known for its tumor‐promoting and immunosuppressive activities. Additionally, ANXA1 could impact the function of T cells and dendritic cells, further attenuating the antitumor immune response and supporting tumor growth and survival. Further research into the interplay between ANXA1 and MHC‐II in breast cancer could elucidate new strategies to counteract the adaptive resistance mechanisms employed by tumors, potentially leading to more effective combination therapies that prevent the upregulation of immune‐inhibitory molecules like ANXA1 in response to PD‐1/PD‐L1 blockade.

ANXA1 expression is significantly higher in TNBC compared to other breast cancer subtypes [31]. The elevated expression of ANXA1 in TNBC may be linked to its multifaceted regulatory roles within the tumor microenvironment. First, ANXA1 is involved in modulating inflammatory responses and immune evasion. Its high expression in TNBC cells may help them escape detection by the immune system, thereby promoting tumor growth and metastasis. Additionally, ANXA1 is associated with EMT, a process crucial for cancer cells to acquire invasive and migratory capabilities. These mechanisms collectively contribute to the aggressive nature of TNBC, highlighting ANXA1 as a potential therapeutic target for this challenging cancer subtype.

Park et al. discovered the association between MHC‐II expression and immune cell infiltration in TNBC [32]. Our study expands on this by exploring several additional dimensions. We delve into the gene expression variations and their functional consequences across different MHC‐II subgroups, using Limma analysis to identify key DEGs such as LTF, MMP8, and LCN2, which are involved in inflammation, immune cell activity, and extracellular matrix remodeling. Additionally, we conducted comprehensive GO and KEGG pathway enrichment analyses, revealing distinct alterations in immune‐related pathways, such as cytokine–cytokine receptor interactions, leukocyte migration, and inflammatory responses, in MHC‐II‐high vs. MHC‐II‐low TNBC cells. Furthermore, we performed MR analysis to investigate the potential causal links between SNPs associated with DEGs and breast cancer risk, as well as the causal role of PD‐L1 expression and its potential link to MHC‐II. This multifaceted approach provides new insights into the immune landscape of TNBC, offering a more detailed understanding of tumor immunity, immune cell migration, and potential therapeutic targets for immunomodulation.

Our findings on ANXA1 and MHC‐II are further supported and enriched by recent research on the pro‐resolving properties of ANXA1 in inflammatory contexts. Specifically, studies have shown that ANXA1, acting through its receptor FPR2, plays a crucial role in mitigating inflammation and promoting immune resolution. For instance, Chen et al. [33] demonstrated that the deficiency of formyl peptide receptor 2 (Fpr2) in myeloid cells exacerbates sepsis‐induced cardiac dysfunction, leading to increased pro‐inflammatory monocyte recruitment and reduced M2‐like macrophages. Critically, their work showed that AnxA1, as an FPR2 agonist, improved cardiac function in septic mice with functional FPR2, but its benefits were limited in KO mice because the FPR2 ligand failed to polarize macrophages to an MHC II‐ phenotype. This directly aligns with our observation of ANXA1's association with MHC‐II subgroups and its potential to modulate immune cell phenotypes [33]. Similarly, Chen et al. [34] showed that AnxA1 alleviated cardiac diastolic dysfunction in mice with inflammatory arthritis. This was achieved, in part, by reducing activated T cells and increasing MHC‐II‐low macrophages infiltration in the heart. These studies collectively suggest that ANXA1's ability to promote an MHC‐II‐low/negative macrophage phenotype is critical for its anti‐inflammatory and pro‐resolving effects. In the context of our breast cancer study, the observed upregulation of ANXA1 in immune cells of PD‐1 nonresponders, coupled with its overall downregulation in breast cancer tissues, could indicate a complex interplay where the tumor microenvironment either co‐opts or is overwhelmed by ANXA1's pro‐resolving signals, potentially contributing to an immunosuppressive milieu despite ANXA1's inherent anti‐inflammatory properties. This highlights a potential adaptive resistance mechanism where the tumor exploits immune regulatory pathways to evade effective antitumor responses [34].

Despite being comprehensive, this study has several limitations that must be acknowledged. First, the associations between ANXA1 expression, MHC‐II co‐expression, and resistance to PD‐1/PD‐L1 therapies are primarily derived from bioinformatics analyses and need further validation in experimental settings. The lack of in vitro and in vivo experimental validation is a significant limitation, as it hinders the translation of our findings into clinical practice. Another limitation is the inherent heterogeneity of breast cancer subtypes, which may affect the generalizability of our findings. Breast cancer encompasses a wide array of biological behaviors and treatment responses, which might not be fully captured by the analyzed data sets. Furthermore, the immunological landscape of breast cancer is complex and influenced by numerous factors beyond ANXA1 and MHC‐II expression, such as the presence of other immune checkpoints, the diversity of the tumor microenvironment, and individual patient variability. Finally, although this study has identified potential biomarkers and mechanisms of resistance to PD‐1/PD‐L1 therapies, further investigation is needed to determine the clinical applicability of these findings. Future studies should encompass a wider range of patient samples, more diverse cancer subtypes, and longitudinal analyses in order to gain a comprehensive understanding of ANXA1 expression dynamics and its impact on immunotherapy outcomes.

## 5 Conclusion

This study underscores the potential role of ANXA1 and its association with MHC‐II in mediating resistance to PD‐1/PD‐L1 blockade in breast cancer. Through comprehensive bioinformatics analyses, we have elucidated the intricate interplay between tumor immunology and therapeutic resistance. Our findings indicate that ANXA1 may serve as a potential marker of adaptive resistance mechanisms. Further research, including validation of its protein expression in cancer cell lines or tissue biopsy samples, is necessary to fully explore its role and to determine whether targeting ANXA1 could enhance the efficacy of immunotherapies. Future studies should focus on validating ANXA1's role through functional and in vivo assays, developing targeted therapies such as small‐molecule inhibitors or monoclonal antibodies, and exploring the synergy of ANXA1‐targeted treatments with existing immunotherapies to improve breast cancer patient outcomes.

## Author Contributions

All Authors had full access to the study data and take responsibility for its integrity, and for the accuracy of the data analysis. Conceptualization: Z.S.J. and Z.S.T. Methodology: J.F.S. Investigation: C.W. Formal analysis: J.F.S. Writing – original draft: H.W. Writing – review and editing: H.W. and C.W. Supervision: Z.S.J. and Z.S.T.

## Conflicts of Interest

The authors declare no conflicts of interest.

## Data Availability

The data used in this paper are from the open source databases GEO and UK Biobank, and the specific analysis data can be obtained from the corresponding author.

## References

[1] M. Nedeljković and A. Damjanović , “Mechanisms of Chemotherapy Resistance in Triple‐Negative Breast Cancer—How We Can Rise to the Challenge,” Cells 8, no. 9 (2019): 957, 10.3390/cells8090957. 31443516 PMC6770896

[2] X. Q. Wang , E. Danenberg , C. S. Huang , et al., “Spatial Predictors of Immunotherapy Response in Triple‐Negative Breast Cancer,” Nature 621, no. 7980 (2023): 868–876, 10.1038/s41586-023-06498-3. 37674077 PMC10533410

[3] P. J. Lei , E. R. Pereira , P. Andersson , et al., “Cancer Cell Plasticity and MHC‐II‐Mediated Immune Tolerance Promote Breast Cancer Metastasis to Lymph Nodes,” Journal of Experimental Medicine 220, no. 9 (2023): e20221847, 10.1084/jem.20221847. 37341991 PMC10286805

[4] E. Kurzejamska , J. Johansson , K. Jirström , et al., “C/EBPβ Expression Is an Independent Predictor of Overall Survival in Breast Cancer Patients by MHC‐II/CD4‐Dependent Mechanism of Metastasis Formation,” Oncogene 3, no. 11 (2014): e125, 10.1038/oncsis.2014.38. PMC4259962 25365481

[5] P. I. Gonzalez‐Ericsson , J. D. Wulfkhule , R. I. Gallagher , et al., “Tumor‐Specific Major Histocompatibility‐II Expression Predicts Benefit to Anti‐PD‐1/L1 Therapy in Patients With HER2‐Negative Primary Breast Cancer,” Clinical Cancer Research 27, no. 19 (2021): 5299–5306, 10.1158/1078-0432.CCR-21-0607. 34315723 PMC8792110

[6] L. Huang , J. Zhang , B. Wei , et al., “Small‐Molecule MHC‐II Inducers Promote Immune Detection and Anti‐Cancer Immunity via Editing Cancer Metabolism,” Cell Chemical Biology 30, no. 9 (2023): 1076–1089.e11, 10.1016/j.chembiol.2023.05.003. 37236192

[7] T. Ma , Y. Tang , T. Wang , et al., “Chronic Pulmonary Bacterial Infection Facilitates Breast Cancer Lung Metastasis by Recruiting Tumor‐Promoting MHC‐IIhi Neutrophils,” Signal Transduction and Targeted Therapy 8, no. 1 (2023): 296, 10.1038/s41392-023-01542-0. 37563136 PMC10415306

[8] D. Kerdidani , E. Aerakis , K. M. Verrou , et al., “Lung Tumor MHC‐II Immunity Depends on In Situ Antigen Presentation by Fibroblasts,” Journal of Experimental Medicine 219, no. 2 (2022): e20210815, 10.1084/jem.20210815. 35029648 PMC8764966

[9] M. E. Ritchie , B. Phipson , D. Wu , et al., “limma Powers Differential Expression Analyses for RNA‐Sequencing and Microarray Studies,” Nucleic Acids Research 43, no. 7 (2015): e47, 10.1093/nar/gkv007. 25605792 PMC4402510

[10] C. Sudlow , J. Gallacher , N. Allen , et al., “UK Biobank: An Open Access Resource for Identifying the Causes of a Wide Range of Complex Diseases of Middle and Old Age,” PLoS Medicine 12, no. 3 (2015): e1001779, 10.1371/journal.pmed.1001779. 25826379 PMC4380465

[11] R. F. Hillary , D. Trejo‐Banos , A. Kousathanas , et al., “Multi‐Method Genome‐ and Epigenome‐Wide Studies of Inflammatory Protein Levels in Healthy Older Adults,” Genome Medicine 12, no. 1 (2020): 60, 10.1186/s13073-020-00754-1. 32641083 PMC7346642

[12] S. L. Shiao , K. H. Gouin, 3rd , N. Ing , et al., “Single‐Cell and Spatial Profiling Identify Three Response Trajectories to Pembrolizumab and Radiation Therapy in Triple Negative Breast Cancer,” Cancer Cell 42, no. 1 (2024): 70–84.e8, 10.1016/j.ccell.2023.12.012. 38194915

[13] G. Yu and Q. Y. He , “ReactomePA: An R/Bioconductor Package for Reactome Pathway Analysis and Visualization,” Molecular BioSystems 12, no. 2 (2016): 477–479, 10.1039/c5mb00663e. 26661513

[14] M. D. Robinson , D. J. McCarthy , and G. K. Smyth , “edgeR: A Bioconductor Package for Differential Expression Analysis of Digital Gene Expression Data,” Bioinformatics 26, no. 1 (2010): 139–140, 10.1093/bioinformatics/btp616. 19910308 PMC2796818

[15] R. Magno and A. T. Maia , “gwasrapidd: An R Package to Query, Download and Wrangle GWAS Catalog Data,” Bioinformatics 36, no. 2 (2020): 649–650, 10.1093/bioinformatics/btz605. 31373609 PMC9883700

[16] C. Minelli , M. F. Del Greco , D. A. van der Plaat , J. Bowden , N. A. Sheehan , and J. Thompson , “The Use of Two‐Sample Methods for Mendelian Randomization Analyses on Single Large Datasets,” International Journal of Epidemiology 50, no. 5 (2021): 1651–1659, 10.1093/ije/dyab084. 33899104 PMC8580269

[17] S. Purcell , B. Neale , K. Todd‐Brown , et al., “PLINK: A Tool Set for Whole‐Genome Association and Population‐Based Linkage Analyses,” American Journal of Human Genetics 81, no. 3 (2007): 559–575, 10.1086/519795. 17701901 PMC1950838

[18] F. A. Wolf , P. Angerer , and F. J. Theis , “SCANPY: Large‐Scale Single‐Cell Gene Expression Data Analysis,” Genome Biology 19, no. 1 (2018): 15, 10.1186/s13059-017-1382-0. 29409532 PMC5802054

[19] C. Domínguez Conde , C. Xu , L. B. Jarvis , et al., “Cross‐Tissue Immune Cell Analysis Reveals Tissue‐Specific Features in Humans,” Science 376, no. 6594 (2022): eabl5197, 10.1126/science.abl5197. 35549406 PMC7612735

[20] A. C. Huang , M. A. Postow , R. J. Orlowski , et al., “T‐Cell Invigoration to Tumour Burden Ratio Associated With Anti‐PD‐1 Response,” Nature 545, no. 7652 (2017): 60–65, 10.1038/nature22079. 28397821 PMC5554367

[21] S. M. Al‐Bahlani , R. M. Al‐Rashdi , S. Kumar , S. S. Al‐Sinawi , M. A. Al‐Bahri , and A. A. Shalaby , “Calpain‐1 Expression in Triple‐Negative Breast Cancer: A Potential Prognostic Factor Independent of the Proliferative/Apoptotic Index,” BioMed Research International 2017 (2017): 9290425, 10.1155/2017/9290425. 28536704 PMC5425834

[22] S. M. Al‐Bahlani , K. H. Al‐Bulushi , Z. M. Al‐Alawi , N. Y. Al‐Abri , Z. R. Al‐Hadidi , and S. S. Al‐Rawahi , “Cisplatin Induces Apoptosis Through the Endoplasmic Reticulum‐Mediated, Calpain 1 Pathway in Triple‐Negative Breast Cancer Cells,” Clinical Breast Cancer 17, no. 3 (2017): e103–e112, 10.1016/j.clbc.2016.12.001. 28089626

[23] L. Rodríguez‐Fernández , I. Ferrer‐Vicens , C. García , et al., “Isoform‐Specific Function of Calpains in Cell Adhesion Disruption: Studies in Postlactational Mammary Gland and Breast Cancer,” Biochemical Journal 473, no. 18 (2016): 2893–2909, 10.1042/BCJ20160198. 27402795

[24] S. Grieve , Y. Gao , C. Hall , J. Hu , and P. A. Greer , “Calpain Genetic Disruption and HSP90 Inhibition Combine to Attenuate Mammary Tumorigenesis,” Molecular and Cellular Biology 36, no. 15 (2016): 2078–2088, 10.1128/MCB.01062-15. 27215381 PMC4946432

[25] X. Pu , S. J. Storr , N. S. Ahmad , et al., “Calpain‐1 Is Associated With Adverse Relapse Free Survival in Breast Cancer: A Confirmatory Study,” Histopathology 68, no. 7 (2016): 1021–1029, 10.1111/his.12896. 26496999

[26] S. K. Lee , Y. Hwang , J. H. Han , S. Haam , H. W. Lee , and Y. W. Koh , “Characteristics of the Immune Microenvironment Associated With RRM2 Expression and Its Application to PD‐L1/PD‐1 Inhibitors in Lung Adenocarcinoma,” American Journal of Cancer Research 13, no. 11 (2023): 5443–5454. 38058821 PMC10695782

[27] D. Xiao , T. Zeng , W. Zhu , et al., “ANXA1 Promotes Tumor Immune Evasion by Binding PARP1 and Upregulating Stat3‐Induced Expression of PD‐L1 in Multiple Cancers,” Cancer Immunology Research 11, no. 10 (2023): 1367–1383, 10.1158/2326-6066. 37566399

[28] C. Shen , S. Zhang , Z. Zhang , et al., “Pan‐Cancer Evidence of Prognosis, Immune Infiltration, and Immunotherapy Efficacy for Annexin Family Using Multi‐Omics Data,” Functional & Integrative Genomics 23, no. 3 (2023): 211, 10.1007/s10142-023-01106-z. 37358720

[29] Z. Z. Yu , Y. Y. Liu , W. Zhu , et al., “ANXA1‐Derived Peptide for Targeting PD‐L1 Degradation Inhibits Tumor Immune Evasion in Multiple Cancers,” Journal for Immunotherapy of Cancer 11, no. 3 (2023): e006345, 10.1136/jitc-2022-006345. 37001908 PMC10069584

[30] W. Xiong , B. Zhang , H. Yu , et al., “RRM2 Regulates Sensitivity to Sunitinib and PD‐1 Blockade in Renal Cancer by Stabilizing ANXA1 and Activating the AKT Pathway,” Advanced Science 8, no. 18 (2021): e2100881, 10.1002/advs.202100881. 34319001 PMC8456228

[31] L. Vecchi , S. T. S. Mota , M. A. P. Zóia , et al., “Interleukin‐6 Signaling in Triple Negative Breast Cancer Cells Elicits the Annexin A1/Formyl Peptide Receptor 1 Axis and Affects the Tumor Microenvironment,” Cells 11, no. 10 (2022): 1705, 10.3390/cells11101705. 35626741 PMC9139391

[32] I. A. Park , S. H. Hwang , I. H. Song , et al., “Expression of the MHC Class II in Triple‐Negative Breast Cancer Is Associated With Tumor‐Infiltrating Lymphocytes and Interferon Signaling,” PLoS One 12, no. 8 (2017): e0182786, 10.1371/journal.pone.0182786. 28817603 PMC5560630

[33] J. Chen , S. Austin‐Williams , C. E. O'Riordan , et al., “Formyl Peptide Receptor Type 2 Deficiency in Myeloid Cells Amplifies Sepsis‐Induced Cardiac Dysfunction,” Journal of Innate Immunity 15, no. 1 (2023): 548–561, 10.1159/000530284. 37068475 PMC10315071

[34] J. Chen , L. V. Norling , J. G. Mesa , et al., “Annexin A1 Attenuates Cardiac Diastolic Dysfunction in Mice With Inflammatory Arthritis,” Proceedings of the National Academy of Sciences of the United States of America 118, no. 38 (2021): e2020385118, 10.1073/pnas.2020385118. 34526398 PMC8463875

